# Spatial genetic structure in *Beta vulgaris* subsp. *maritima* and *Beta macrocarpa* reveals the effect of contrasting mating system, influence of marine currents, and footprints of postglacial recolonization routes

**DOI:** 10.1002/ece3.1061

**Published:** 2014-04-18

**Authors:** Marie Leys, Eric J Petit, Yasmina El-Bahloul, Camille Liso, Sylvain Fournet, Jean-François Arnaud

**Affiliations:** 1 Laboratoire de Génétique et Évolution des Populations Végétales, UMR CNRS 8198, Bâtiment SN2, Université des Sciences et Technologies de Lille – Lille 1Villeneuve d'Ascq Cedex, F-59655, France; 2 UMR CNRS 6553 ECOBIO, Station biologique, Université de Rennes 1Paimpont, F-35380, France; 3 Unité d'Amélioration des Plantes Conservation et Valorisation des Ressources Phytogénétiques, Centre Régional de la Recherche Agronomique de Rabat, INRA-MarocRabat-Instituts, 10101, Morocco; 4 UMR 1349 IGEPP, INRA – Agrocampus Ouest-Université de Rennes 1Bât 320, BP35327, Le Rheu Cedex, 35653, France

**Keywords:** Cytonuclear genetic diversity, glacial refugia, marine currents, postglacial expansion, spatial genetics

## Abstract

Understanding the factors that contribute to population genetic divergence across a species' range is a long-standing goal in evolutionary biology and ecological genetics. We examined the relative importance of historical and ecological features in shaping the present-day spatial patterns of genetic structure in two related plant species, *Beta vulgaris* subsp. *maritima* and *Beta macrocarpa*. Using nuclear and mitochondrial markers, we surveyed 93 populations from Brittany (France) to Morocco – the southern limit of their species' range distribution. Whereas *B. macrocarpa* showed a genotypic structure and a high level of genetic differentiation indicative of selfing, the population genetic structure of *B. vulgaris* subsp. *maritima* was consistent with an outcrossing mating system. We further showed (1) a strong geographic clustering in coastal *B. vulgaris* subsp. *maritima* populations that highlighted the influence of marine currents in shaping different lineages and (2) a peculiar genetic structure of inland *B. vulgaris* subsp. *maritima* populations that could indicate the admixture of distinct evolutionary lineages and recent expansions associated with anthropogenic disturbances. Spatial patterns of nuclear diversity and differentiation also supported a stepwise recolonization of Europe from Atlantic-Mediterranean refugia after the last glacial period, with leading-edge expansions. However, cytoplasmic diversity was not impacted by postglacial recolonization: stochastic long-distance seed dispersal mediated by major oceanic currents may mitigate the common patterns of reduced cytoplasmic diversity observed for edge populations. Overall, the patterns we documented here challenge the general view of reduced genetic diversity at the edge of a species' range distribution and provide clues for understanding how life-history and major geographic features interact to shape the distribution of genetic diversity.

## Introduction

Plant populations are often composed of distinct breeding units, and patterns of genetic variation within and among populations are determined primarily by mating system and the neutral microevolutionary process of gene flow (Hamrick and Godt 1996; Duminil et al. 2009). Historical events such as Pleistocene climatic changes have also played a fundamental role in generating phylogeographic structure within species' ranges (e.g., Hewitt 2004). In the northern hemisphere, the alternation of Quaternary glacial and interglacial episodes resulted in substantial changes in species' distribution. These events have left footprints in their genetic structure and may explain the common poleward decrease in genetic diversity exhibited by terrestrial taxa (Hewitt 1999).

From the extensive body of phylogeographic work on European temperate species, it is acknowledged that the Mediterranean Basin acted as a major refugium during these glacial cycles. In particular, the Iberian, Italian, and Balkan Peninsulas have been considered as differentiation centers and origins of postglacial northward expansion (Hewitt 2000). However, the literature remains largely biased with respect to geographic coverage and the type of organism studied. In contrast to Western Europe, North Africa is largely underrepresented. This imbalance is unfortunate, because North Africa harbors hot spots of plant biodiversity and endemism (Médail and Quézel 1997). Furthermore, most of the studies conducted in North Africa have focused on inland woody species (e.g., Lumaret et al. 2002; Migliore et al. 2012) rather than on coastal plants (Ortiz et al. 2007). Coastal plant species offer, however, ideal case studies for interpreting phylogeographic patterns. They have a very wide latitudinal and longitudinal range, but are restricted to a narrow belt along the shoreline, suggesting limited migration routes and/or postglacial recolonization pathways. In addition to climatic oscillations, the regional distribution of coastal plant species may also be influenced by marine currents *via* seed drift (dispersal), leading to sharp genetic discontinuities or nonintuitive genetic affinities among populations (Kadereit et al. 2005).

Beyond the influence of historical events, patterns of genetic structure across a species range are also informative about contemporary processes. Current spatiotemporal dynamics of plant species' distribution may indeed be influenced by anthropogenic disturbance, acting as dispersal agents through exchanges or interfering with natural recolonization by modifying the landscape (D'Hondt et al. 2012). Human impact on the environment is a long-standing feature of the Mediterranean Basin (Blondel 2006). For example, the long history of agriculture and other ancient and recent human activities have resulted in an evolving landscape mosaic, with land uses changing in space and time. Therefore, population-level processes are highly complex, and spatial genetic structure is thus tightly coupled to ongoing processes that superimpose new signatures on existing natural phylogeographic patterns. The interplay between mating system, geographic distribution, oceanographic features/sea circulation, past climatic changes, and anthropogenic pressures is likely responsible for the current genetic diversity and species distribution of coastal plants along the southern Atlantic coast and the Mediterranean Basin (Rodríguez-Sánchez et al. 2008).

The present study set out to depict large-scale patterns of cytoplasmic and nuclear genetic structure in two related species thought to have contrasting mating systems: the wild sea beet (*Beta vulgaris* subsp. *maritima*) and its close relative, *Beta macrocarpa*. Sea beet is a typical coastal taxon, mainly outcrossing, with a wide distribution from the Canary Islands in the west, northward along Europe's Atlantic coast and Baltic Seas (Biancardi et al. 2012). Sea beet also extends eastward through the Mediterranean Basin along the coastline but also in inland man-made habitats (Desplanque et al. 1999; Arnaud et al. 2009). *B. macrocarpa*, a self-compatible species, has a more restricted geographic distribution located in inland or coastal habitats in western and eastern Mediterranean areas (Buttler 1977; Letschert 1993; Castro et al. 2013). Elucidating the underlying mechanisms of range shifts and the determinants of population establishment are fundamental questions, especially in the context of climate change. Based on a comprehensive sampling in Western Europe and in North Africa and using nuclear and cytoplasmic data, we thus applied classical *F*-statistics, Bayesian clustering, and spatial multivariate methods to depict the phylogeographic and genetic structure of both species. Specifically, we asked the following questions:

1. Do *B. vulgaris* subsp. *maritima* and *B. macrocarpa* show significant genetic differentiation and different genotypic structure indicative of contrasting mating system, as suggested by previous studies based on greenhouse experiments and allozyme survey?
2. What are the roles of alternative evolutionary processes in shaping different lineages along the southern European/northern African distribution of these two species? We hypothesized that range shift driven by last Quaternary glaciation, ocean circulation patterns, and anthropogenic disturbances could be fundamental processes explaining large-scale patterns of genetic diversity and divergence we observed.

## Materials and Methods

### The species

**Figure 1:**
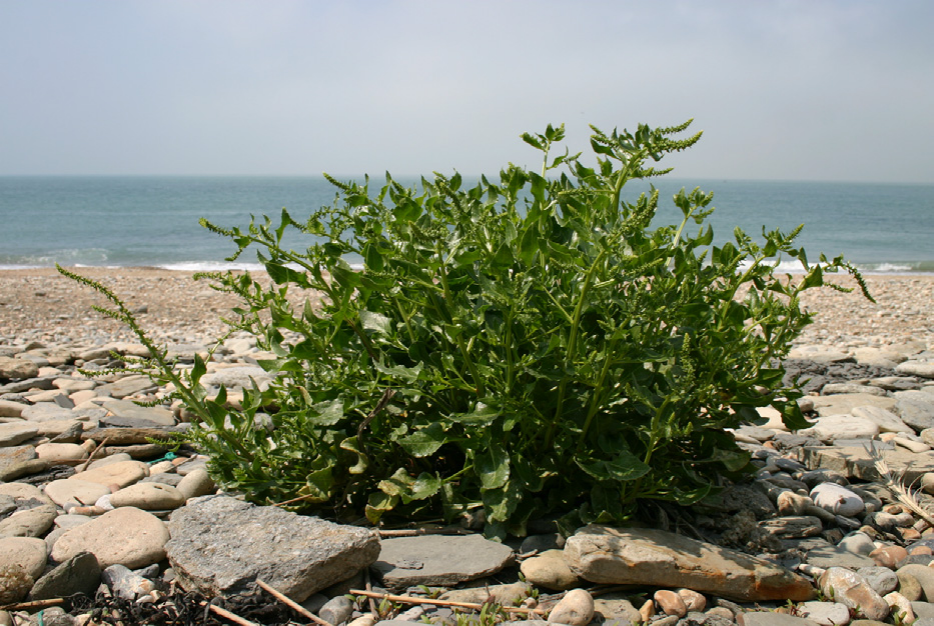
Wild sea beet (*Beta vulgaris* subsp. *maritima*), the wild ancestor of all cultivated beets. Sea beet is a typical coastal taxon growing in the supralittoral fringe, typically on beaches of sand or pebbles or associated with rocky substrates.

The genus *Beta* is divided into two sections, that is, the *Beta* section and the *Corollinae* section (Biancardi et al. 2012). The *Beta* section includes five taxa: (1) the sea beet (*Beta vulgaris* subsp. *maritima*, see Fig. 1), which is considered as the wild ancestor of all cultivated beets; (2) the different forms of cultivated beets (*Beta vulgaris* subsp. *vulgaris*), including fodder, leaf, or sugar beets; (3) *Beta macrocarpa*, which is an annual self-compatible plant thought to reproduce predominantly by autogamy; (4) *Beta patula*, which is endemic to an islet of the Madeira Archipelago; and (5) *Beta vulgaris* subsp. *adanensis*, a self-compatible species that is only found in Eastern Mediterranean areas, Greece and Turkey (Frese et al. 1990; Kadereit et al. 2006; Biancardi et al. 2012). Therefore, in our extensive sampling area in the western Mediterranean, only two species of the *Beta* section can be found in coastal and inland ruderal habitats: *Beta vulgaris* subsp. *maritima* and *Beta macrocarpa*.

Sea beet is a short-lived perennial species growing in the supralittoral fringe (Fig. 1), typically on beaches of sand or pebbles, or associated with rocky substrates such as cliffs overhanging the sea (De Cauwer et al. 2010). These ecosystems are characterized by linear physical boundaries, increased salinity, periodic strong winds, and soil instability due to water movements during the highest tides. In addition, a particular wild form of *B. vulgaris* subsp. *maritima* has colonized human-disturbed inland habitats in the western part of the Mediterranean area (Desplanque et al. 1999). These inland ruderal beets have founded populations associated with past and present road works (roadsides, car parks), wastelands, rubble deposits, or gardens (Arnaud et al. 2009). Sea beet is a diploid (2*n* = 18) gynodioecious species, has an outcrossing mating system – enforced by a gametophytic self-incompatibility system with up to four gametophytic S loci – and relies on wind pollination (Owen 1942). No vegetative reproduction occurs, and dispersal is only mediated by seed and/or pollen movements. Seeds are clustered in an irregular dry fruit, forming a multigerm seedball composed of one to eight seeds (De Cauwer et al. 2011). Although this seedball is mainly dispersed by gravity with a large proportion of seeds falling in the vicinity of the mother plant (Arnaud et al. 2011; De Cauwer et al. 2012), it has a corklike layer around the seeds, which may confer buoyancy and make it amenable to dispersal in seawater (see Fievet et al. 2007).

Only flowering characteristics such as tepal morphology allow distinguishing between *B. v*. subsp. *maritima* and *B*. *macrocarpa*. *B. macrocarpa* is a self-compatible species thought to be predominantly self-fertilizing (Jung et al. 1993; Bruun et al. 1995). Nonmorphologically distinguishable diploid (*2n* = 18) and tetraploid (2*n* = 36) cytotypes occur in *B. macrocarpa* (Castro et al. 2013). Using diverse accessions of western Mediterranean areas, both cytotypes were found to be annual and fully self-compatible (Buttler 1977; Lange and Debock 1989). Letschert (1993), Bartsch and Ellstrand (1999), and Villain (2007) provided some evidence suggesting nearly complete autogamy in both diploid and tetraploid accessions of *B. macrocarpa*.

### Study area and sampling

**Figure 2:**
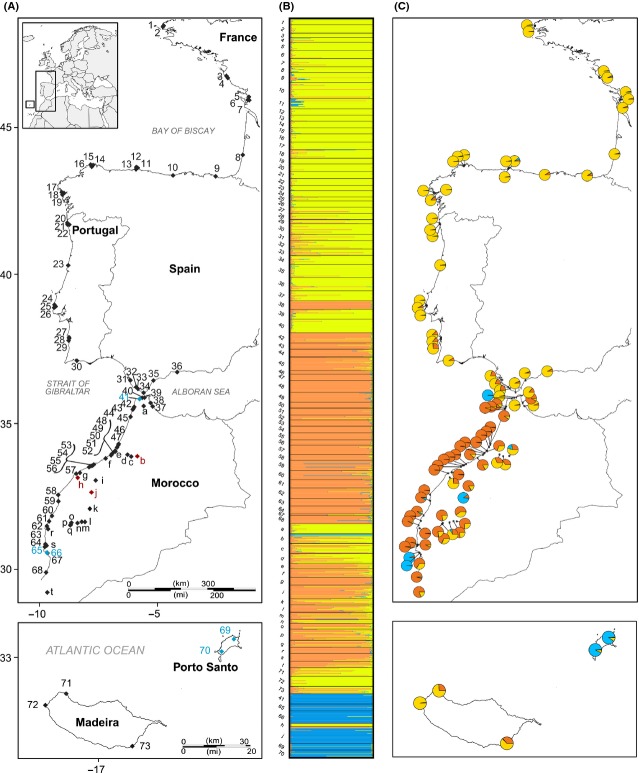
(A) Map of the geographic location of the 93 sampled stations for *Beta vulgaris* subsp. *maritima* and *Beta macrocarpa*. Each *B. vulgaris* subsp. *maritima* station is represented by a black diamond. *B. macrocarpa* stations are symbolized by blue diamonds and stations composed of a mixture of both species by red diamonds. (B) Bayesian clustering following Pritchard et al. (2000). Assignment probabilities of individual memberships of *B. vulgaris* subsp. *maritima* and *B. macrocarpa* into three inferred clusters for the first modal value (*K* = 3). Each individual is represented by a thin horizontal line (*y* axis) partitioned into three colored segments that represent the individual's estimated membership fractions to the *K* = 3 clusters (*x*-axis). (C) Map of mean population membership probabilities for the three clusters.

We sampled 93 localities distributed along the eastern Atlantic coast, from Brittany, France, to southern Morocco, but also in the Mediterranean region on both sides of the Strait of Gibraltar (Fig. 2A). In Western Europe, samples were collected at 36 sites and labelled from 1 to *36*. In Morocco, 52 coastal and inland localities were sampled, among which 32 sites were representative of typical coastal populations collected along the shoreline (labelled from 37 to 68, Fig. 2A) and 20 were from inland ruderal sites (labelled from *a* to *t*, Fig. 2A). Five sites in the Madeira archipelago were also sampled and numbered from 69 to 73. In total, 1816 individuals were collected with an average sample size per site of 19.53 (SE ± 6.07). The population labelled 67 was made up of only four individuals and was not included in analyses dealing with mean levels of genetic diversity, such as the estimation of allelic richness. Sampling was carried out during five successive surveys between 2007 and 2011. Fresh leaves from the collected individuals were dried and stored in silica gel at room temperature until DNA extraction. Appendix 1 provides full details on the sampling localities.

### Genetic data collection

Extraction and purification of total DNA were performed using a DNeasy 96 Plant Kit following the standard protocol for isolation of DNA from plant leaf tissue outlined in the DNeasy 96 Plant protocol handbook (QIAGEN Inc., Courtaboeuf, France).

#### Nuclear microsatellites

All individuals were examined for nuclear genetic variation using eight microsatellite loci, named *Bvm3* (Mörchen et al. 1996), *Gtt1*, *Caa1* (Viard et al. 2002), *Sb04*, *Sb06*, *Sb07*, *Sb15* (Richards et al. 2004), and *FDSB1027* (McGrath et al. 2007). Polymerase chain reaction (PCR) conditions and allele-sizing procedures can be found in Fénart et al. (2007). Electrophoresis and genotyping were performed on an ABI automated sequencer model 3110 (Applied Biosystems Inc.) as described in De Cauwer et al. (2010). To minimize the rate of genotyping errors, a second round of PCR and electrophoresis were performed for individuals with dubious multilocus genotypes, that is, with missing data or displaying rare alleles.

#### Mitochondrial minisatellites

To gain information on cytoplasmic genetic diversity, mitochondrial polymorphism was characterized by genotyping all individuals at four mitochondrial minisatellite loci: *TR1*, *TR2*, *TR3*, and *TR4* (Nishizawa et al. 2000). PCR amplifications, cycling conditions, and genotyping were performed according to protocols previously described in Fénart et al. (2008). As the mitochondrial genome is maternally inherited as a single linkage unit, each genotype combination for these four minisatellite loci was analyzed as a single haplotype for the levels of cytoplasmic diversity and population differentiation, as in De Cauwer et al. (2012).

### Statistical analyses of cytoplasmic and nuclear genetic variation

#### Distinguishing between *B. vulgaris* subsp*. maritima* and *B. macrocarpa*

As pointed out by Buttler (1977), only tepal characters are relevant criteria to distinguish between *B. v*. subsp. *maritima* and *B. macrocarpa*. However, sampling was carried out at the beginning of the flowering season, when *B. vulgaris* subsp. *maritima* shows large phenotypic plasticity and is morphologically similar to *B*. *macrocarpa*. However, as *B. macrocarpa* is a self-fertilizing species, the detection of multilocus fixed-homozygote genotypes can help to determine a posteriori which individuals belong to *B. macrocarpa*. Because (1) the two species may possibly hybridize (Kishima et al. 1987; Lange and Debock 1989; Bartsch and Ellstrand 1999) and (2) *B. macrocarpa* tetraploid forms with fixed heterozygosity can occur (Letschert 1993), we also discriminated individuals on the basis of their probability of belonging to one or the other species due to allele frequency differences in multilocus genotypes. To do so, we used the model-based Bayesian algorithm implemented in STRUCTURE and described in Pritchard et al. (2000). Bayesian clustering assumes that each individual has admixed ancestral origins in gene pools that have correlated allele frequencies because of migration or shared ancestry (Falush et al. 2003). Using the whole data set, we assessed the number of potential clusters (*K*) from 30 runs along a range of *K* varying from 1 to 50 with 2.10^6^ Markov chain Monte Carlo replications following a burn-in period of 100,000 iterations without any prior information on the putative population affiliation of individuals. Among the 30 replicates, 15 with the highest likelihood were retained for subsequent analyses. The ad hoc statistic *ΔK* was then calculated to determine the most accurate number of *K* clusters (Evanno et al. 2005).

The Bayesian clustering described in Pritchard et al. (2000) assigns individuals by creating groups within which Hardy–Weinberg (HW) disequilibrium and linkage disequilibrium (LD) are minimized. Nonetheless, inbreeding may induce departures from HW expectations and LD among loci, which can lead to bias or erroneous inferences. To circumvent this, we ran two parallel extensive runs by keeping or removing *B. macrocarpa* individuals to verify the consistency of clustering results.

Additional analyses were conducted using spatially explicit Bayesian clustering, that is, incorporating spatial trends and autocorrelation in the prior distribution on individual admixture coefficients, as described in Chen et al. (2007) and Durand et al. (2009). Using TESS version 2.3 (Grenoble INP -TIMC-IMAG, Faculty of Medicine, La Tronche, France), we ran 1.10^6^ (100,000 burn-in) MCMC iterations from *K* = 1 to *K* = 20 (25 replicates per *K* value) using the BYM admixture model described in Durand et al. (2009) and the geographic distances between individuals. Of the 25 replicates per *K* tested, 20 with the lowest deviance information criterion (DIC) were retained. We set the Dirichlet parameter of the allele frequency model, the trend degree to 1.0, and the admixture and spatial interaction parameters to default values. To reveal which *K* may provide the best fit to the genetic data, average DIC values were plotted against *K*.

Similarity coefficients between runs and the average matrices of individual membership proportions were estimated using CLUMPP version 1.1.2 (Jakobsson and Rosenberg 2007). Clusters were displayed using DISTRUCT version 1.1 (Rosenberg 2004).

#### Basic parameters of genetic diversity

Genetic variation was examined per locus and per population with descriptive statistics: the total and mean number of alleles (*A*_*t*_ and *A*_*n*_, respectively), allelic richness (*A*_r_), and level of gene diversity (*H*_E_), all using FSTAT version 2.9.3.2 (Goudet 1995). Allelic richness was estimated using the rarefaction approach proposed by El Mousadik and Petit (1996) and based on a minimal population size of eight diploid individuals. For additional information on population genetic uniqueness, estimates of private allelic richness (*A*_r_*P*) were computed following a rarefaction procedure (*n* = 8) using ADZE software (Szpiech et al. 2008).

Genotypic linkage disequilibrium among all locus-pair combinations was assessed prior to other analyses using a Markov chain approximation of the Fisher's exact test, based on the contingency tables for all pairs of loci in each population, as implemented in GENEPOP version 4.0.1.1 (Raymond and Rousset 1995). Departures from HW equilibrium for each microsatellite locus and overall loci were quantified using the intrapopulation fixation index (*F*_IS_). Statistical significance of *F*_IS_ values was subsequently assessed using the randomization procedure provided by FSTAT. Sequential Bonferroni corrections for multiple comparisons were applied following Rice (1989).

Levels of genetic variation (*A*_r_, *A*_r_*P*, and *H*_E_) were compared between the genetically distinct groups depicted through Bayesian clustering and multivariate analyses described later. Statistical significance of differences was tested using a one-way analysis of variance followed by Tukey's multiple-comparisons test. To assess whether a northward-declining diversity gradient occurred in *B. vulgaris* subsp. *maritima*, linear regressions were applied on multilocus genetic diversity estimates and spatial attributes of populations: latitude and geographic distance measured along the coastline from the most southern sampling site. Linear regressions were performed on separate population data sets to take into account the presence of genetic discontinuities. A potential bias could arise because levels of allelic diversity often varied among the loci. Therefore, we also performed linear mixed models in analyzing genetic diversity as a function of geographic locations, with loci as random intercepts.

#### Extent of genetic differentiation

We investigated population structure using classical *F*-statistics according to Weir and Cockerham (1984) to test for population differentiation across all populations, using FSTAT. Similarly to *F*_IS_ values, we used a permutation test to determine whether observed values of *F*_ST_ were significantly different from 0 for each locus and overall loci (10,000 randomizations of multilocus genotypes among populations). Furthermore, we compared the mean pairwise genetic differentiation between genetic clusters following the statistical procedure accounting for nonindependent data described in Coulon et al. (2006). The population grouping corresponded to the genetic clusters depicted through Bayesian clustering and multivariate analyses described later. The apportionment of genetic variation was also assessed following the hierarchical procedure proposed by Yang (1998). Hierarchical *F*-statistics were carried out within and among genetically distinct groups depicted by clustering and multivariate analyses described later. Using the R package HIERFSTAT (Goudet 2005), the significance of genetic differentiation among populations was assessed by randomly permuting individuals (10,000 permutations) among populations within groups. Genetic differentiation among genetic groups was tested by carrying out 10,000 permutations of populations among genetic groups.

#### Spatial multivariate analysis

We also analyzed our data set using a spatially explicit multivariate method: the spatial principal component analysis (sPCA) described in Jombart et al. (2008). The advantage of sPCA is that it imposes no genetic assumptions on mating system, population structure, or allele frequency models (Jombart et al. 2009). Basically, the method aims to find independent synthetic variables that maximize the product of the spatial autocorrelation calculated on a set of allelic frequencies using Moran's index and the genetic variance among individuals or populations. Spatial information used for the computation of Moran's *I* is stored in a spatial weighting binary matrix determined through a neighborhood graph. The Gabriel graph was selected to perform the sPCA because it showed the best fit with our genetic data (see Table S1). To evaluate the consistency of spatial patterns observed, the significance of global and local spatial structures was assessed using permutation tests as described in Jombart et al. (2008). Main results provide a geographic map of entity scores allowing a visual assessment of spatial genetic structure. To draw a comprehensive synthetic representation, each of the first three principal component scores was simultaneously represented into a channel of colors as in Menozzi et al. (1978). All the analyses were performed using the ADEGENET package implemented in R (Jombart 2008; R Development Core Team 2011).

#### Analysis of isolation-by-distance patterns

We studied the relationships between genetic divergence and spatial isolation of populations using unidirectional Mantel correlograms (Oden and Sokal 1986; Arnaud et al. 2009). Genetic divergence was quantitated using the *D*_CE_ chord distance (Cavalli-Sforza and Edwards 1967). These analyses were used to compare the strength of spatial structuring as depicted by nuclear and cytoplasmic data. Mantel correlograms were designed using the normalized Mantel statistics (*rz*, Smouse et al. 1986) following the procedure described in Oden and Sokal (1986). For each distance class, designed with a minimum of 15 pairwise comparisons, the significance of *rz* values was tested with a Mantel test (10,000 permutations). All analyses were performed using PASSAGE version 1.1.2.3 (Rosenberg and Anderson 2011).

## Results

### Distinguishing between *B. vulgaris* subsp. *maritima* and *B. macrocarpa*

The *ΔK* statistic showed a strong mode at *K* = 3: Bayesian clustering analysis separated the whole data set into three genetically distinct groups (Figs. 2B,C, S1). The depicted clusters corresponded to (1) European sea beet populations also including some Moroccan populations located near Gibraltar; (2) the remaining Moroccan sea beet populations; and (3) all *B. macrocarpa* populations. We are very confident that the three depicted genetic clusters match *B*. *vulgaris* subsp. *maritima* and *B. macrocarpa* delimitations. Indeed, along with particular multilocus genotypes found in the third cluster for which we hypothesize to be exclusively composed of *B. macrocarpa* individuals, the hierarchical nuclear genetic differentiation between the two species based on this clustering pattern was the strongest and highly significant (*F*_CT_ = 0.288, *P* < 0.001). Among the two groups of sea beets, a significant hierarchical differentiation supported this clustering: Genetic differentiation among populations within these two clusters accounted for most of the genetic variability (*F*_PR_ = 0.131; *P* < 0.001), and the differentiation between the two clusters, although weaker, was highly significant (*F*_RT_ = 0.039; *P* < 0.001). The *K* versus *ΔK* distribution was multimodal with another mode at *K* = 6 suggesting further hierarchical structure within the sampled area (Figs. S1, S2). The similarity coefficients across independent runs were all larger than 0.998, which suggests the absence of genuine multimodality across runs. Inbreeding did not induce any bias because an identical clustering pattern was found when only analyzing *B. vulgaris* subsp. *maritima* populations (Fig. S2). Assisting the Bayesian clustering using spatial distribution of individuals yielded identical results but with lower statistical support: five *B*. *vulgaris* subsp. *maritima* genetic clusters were also inferred in TESS analyses, but similarity coefficients among runs were considerably low, indicating genuine multimodality (*H′* = 0.599, from 0.457 to 0.737; see Fig. S3).

Overall, based on genotypic structure and on the extensive runs of Bayesian clustering analyses, eight Moroccan sites and two localities on Santo Porto island were composed of *B. macrocarpa* individuals and of an admixture of *B. macrocarpa* and *B. vulgaris* subsp. *maritima* individuals (sites labelled *41*, *65*, *66*, *b*, *h*, *j*, *69*, and *70*, see Fig. 2 and Appendix). Although in sympatry, no admixture indicative of hybridization was observed between the two species.

### Genotypic linkage, Hardy–Weinberg disequilibrium, and levels of nuclear and cytoplasmic polymorphism

Within each *B. vulgaris* subsp. *maritima* population, exact tests for LD between microsatellite loci showed only 31 significant *P*-values of 2380 comparisons (1.30%) after Bonferroni correction (119 expected from type I error at *α* = 0.05). LD was only found in 13 of the 85 *B. vulgaris* subsp. *maritima* populations and involved different pairs of loci. This suggests spurious LD due to genetic drift and/or population substructuring effects (De Cauwer et al. 2012).

Using multilocus probability tests, we detected significant departures from HW equilibrium in 32 of the 85 populations of *B. vulgaris* subsp. *maritima*. However, only a few of these populations displayed consistent *F*_IS_ values across loci, suggesting departures from panmixia (Appendix). Over all *B. vulgaris* subsp. *maritima* populations, five of eight loci showed significant deviation from HW genotypic proportions, although these *F*_IS_ estimates were very low, ranging from 0.007 to 0.087 (Table 1). Combined probability tests over all loci and across all populations resulted in an overall departure from HW due to only few significant single-locus departures within populations or significant multilocus departures in only a few populations. In contrast, all but two populations of the third cluster defined as corresponding to *B. macrocarpa* exhibited very high, significantly positive, single- and multilocus *F*_IS_ values (Table 1 and Appendix). The only exceptions were the *B. macrocarpa* populations located on Santo Porto Island in Madeira archipelago, one of which (population labelled 70) had a highly negative mean *F*_IS_ value due to fixed heterozygosity at multilocus genotypes (Appendix). The other one was composed of individuals characterized by either fixed homozygosity or fixed heterozygosity.

**Table 1:** Genetic diversity measures and *F*-statistics following Weir and Cockerham (1984) estimated over all *Beta vulgaris* subsp. *maritima* (*n* = 85) and *B. macrocarpa* (*n* = 8) populations for eight nuclear microsatellite and four cytoplasmic minisatellite loci

Locus	*A*_*t*_	*A*_r_	*H*_E_	Allelic size range (bp)	*F*_IT_	*F*_ST_	*F*_IS_
*B. maritima*							
Nuclear							
*FDBS1027*	32	6.426	0.614	170–228	0.184*	0.170*	0.018
*Bvm3*	56	12.043	0.831	093–157	0.195*	0.134*	0.070*
*Caa1*	52	10.059	0.809	128–230	0.205*	0.130*	0.087*
*Gtt1*	10	3.650	0.418	104–127	0.200*	0.177*	0.028
*Sb04*	22	5.320	0.608	167–205	0.223*	0.172*	0.062*
*Sb06*	14	6.675	0.699	145–183	0.198*	0.168*	0.037*
*Sb07*	44	11.505	0.827	240–286	0.181*	0.127*	0.062*
*Sb15*	28	6.622	0.624	131–189	0.132*	0.126*	0.007
Average	32.250	7.788	0.679	–	0.190*	0.148*	0.050*
Cytoplasmic							
*TR1*	15	7.167	–	396–780	–	0.361*	–
*TR2*	3	1.109	–	359–456	–	0.175*	–
*TR3*	4	2.589	–	309–440	–	0.389*	–
*TR4*	7	2.648	–	304–485	–	0.392*	–
Average	7.250	3.37825	–	–	–	–	–
Haplotypes	45	3.573	–	–	–	0.350*	–
*B. macrocarpa*							
Nuclear							
*1027*	8	3.511	0.264	171–201	1.000*	0.688*	1.000*
*Bvm3*	10	3.374	0.219	096–117	0.949*	0.759*	0.786*
*Caa1*	9	3.069	0.180	128–179	0.901*	0.714*	0.655*
*Gtt1*	5	2.794	0.199	107–121	0.601*	0.051	0.580*
*Sb04*	6	2.826	0.192	167–183	0.858*	0.676*	0.561*
*Sb06*	5	2.279	0.167	146–168	0.886*	0.841*	0.279
*Sb07*	13	4.063	0.360	245–269	0.910*	0.300*	0.870*
*Sb15*	6	2.469	0.174	131–169	0.886*	0.842*	0.279
Average	7.750	3.048	0.219	–	0.905*	0.588*	0.769*
Cytoplasmic							
*TR1*	3	2.207	–	494–527	–	0.748*	–
*TR2*	1	1.000	–	359	–	–	–
*TR3*	3	1.210	–	374–440	–	0.873*	–
*TR4*	1	1.000	–	364	–	–	–
Average	2	1.354	–	–	–	–	–
Haplotypes	5	2.073	–	–	–	0.743*	–

*A*_*t*_, total number of alleles; *A*_r_, allelic richness; *H*_E_, gene diversity; *F*_IT_, *F*_ST,_ *F*_IS_: fixation indices following Weir and Cockerham (1984); –, not applicable (haploid data).

Significance of departure from Hardy–Weinberg equilibrium within (*F*_IS_), over all populations (*F*_IT_), and genetic differentiation (*F*_ST_) was tested by 10,000 randomizations (*P < 0.0001). Bonferroni corrections were applied to the mean nuclear *F*_ST_ estimates and cytoplasmic loci. Allelic size ranges in base pairs (bp) are also indicated for each locus.

Nuclear microsatellite loci for *B. vulgaris* subsp. *maritima* populations displayed moderate to high numbers of alleles, ranging from 10 (*Gtt1*) to 56 (*Bvm3*), with an average of 32.25 alleles and a mean gene diversity (*H*_E_) of 0.679 (Table 1). Mitochondrial genetic diversity was much lower, with a number of alleles varying from 3 (*TR2*) to 15 (*TR1*). Based on a single haplotype determined by combining the alleles at four loci, we found a total of 45 haplotypes, of which four represented 77.31% of the total number of haplotypes. In sharp contrast, *B. macrocarpa* displayed much lower levels of genetic diversity, with an average of 7.750 alleles for nuclear variation and a total number of 5 haplotypes for cytoplasmic polymorphism (see Table 1). The geographic distribution of mtDNA haplotypes within populations can be visualized in Fig. S4.

### Geographic trends in the levels of genetic diversity

**Figure 3:**
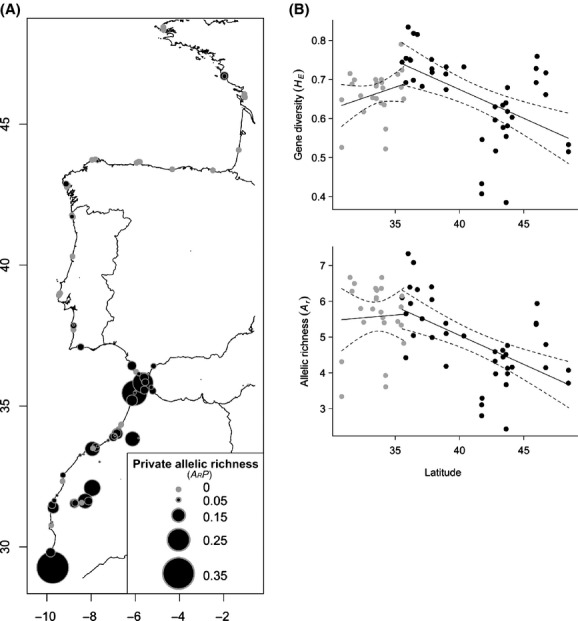
Spatial patterns of genetic diversity. (A) Nuclear private allelic richness (*A*_r_*P*) of *B. vulgaris* subsp. *maritima* populations is proportional to the size of the dot mapped onto the population location. (B) Linear regressions of gene diversity (*H*_E_) and allelic richness (*A*_r_) based on nuclear polymorphism with respect to latitude. Black dots represent genetic diversity values for northern populations, from Brittany to northern Moroccan populations at the Strait of Gibraltar (populations labelled 1–40 in Fig. 1). Gray dots represent values for Moroccan populations at lower latitudes (populations labelled 43 to *t*). Dashed lines indicate the 95% confidence intervals.

Estimates of genetic diversity (*A*_r_, *A*_r_*P*, and *H*_E_) varied greatly among *B. vulgaris* subsp. *maritima* populations (Appendix). A striking northward decrease in private nuclear alleles within populations was observed over the sampled area, with the highest private allelic richness being found on both sides of the Strait of Gibraltar and in Moroccan inland ruderal populations (Fig. 3A). Nuclear gene diversity and allelic richness significantly decreased with latitude from the Gibraltar region (*H*_E_: *r*^2^ = 0.283, *P* < 10^−4^; *A*_r_: *r*^2^ = 0.323, *P* < 10^−4^; Fig. 3B). Similar trends were described using the geographic distance calculated along the coastline (*H*_E_: *r*^2^ = 0.211, *P* < 0.003; *A*_r_: *r*^2^ = 0.242, *P* < 0.001). In contrast, no significant trends were observed for Moroccan *B. vulgaris* subsp. *maritima* populations at lower latitudes (Fig. 3B). Finally, regarding cytoplasmic polymorphism, no significant relationships with latitude or coastline distance were observed for any parameter of genetic diversity based on multilocus data (data not shown). Very similar trends of decreasing nuclear gene diversity with latitude were depicted using linear mixed models with loci as random intercept (see Table S2 and Fig. S5).

We then focused on Morocco, a region considered as a hot spot of genetic diversity, to compare coastal (*C*) and inland ruderal (*R*) populations. Inland ruderal populations exhibited higher levels of gene diversity (*H*_E_, *C*: 0.675, *R*: 0.734; *P* < 10^−3^), allelic richness (*A*_r_, *C*: 5.538, *R*: 6.400; *P* < 10^−3^), and mean number of alleles (*A*_*n*_, *C*: 7.569, *R*: 8.835; *P* < 10^−2^) for nuclear data. Inland ruderal populations also showed a higher level of allelic richness for cytoplasmic data (*A*_r_, *C*: 1.830, *R*: 2.209; *P* < 10^−3^; *A*_r_ *Haplo, C*: 3.349, *R*: 4.532; *P* < 10^−2^). However, no significant differences were observed for private allelic richness (*A*_r_*P*, *C*: 0.066, *R*: 0.093; *P* = 0.255). Regarding genotypic structure, mean multilocus *F*_IS_ values marginally differed between inland ruderal populations and coastal populations (*C*: 0.030, *R*: 0.073; *P =* 0.057), and no difference was observed for nuclear multilocus *F*_ST_ (*C*: 0.115, *R*: 0.074; *P* = 0.112).

### Mean levels of genetic differentiation

A strong genetic differentiation among *B. vulgaris* subsp. *maritima* populations was found using mitochondrial minisatellite markers: The mean *F*_ST_ estimate, based on haplotypes, was 0.350 (*P* < 0.01, Table 1). Highly significant spatial genetic differentiation also occurred for nuclear polymorphism, with consistent single-locus *F*_ST_ estimates ranging from 0.126 to 0.177 (all at *P* < 0.0001) with a mean multilocus *F*_ST_ of 0.148 (*P* < 0.01, see Table 1). Among the 3570 comparisons, mean pairwise *F*_ST_ between populations ranged from 0 to 0.461 (94.64% at *P* < 0.05) and from 0 to 0.973 (44.14% at *P* < 0.05) for nuclear and cytoplasmic data, respectively (Fig. S6). Altogether, these results clearly indicated genetically structured populations for both kinds of markers over the whole sampled area. Among the eight sampled populations of *B. macrocarpa*, nuclear and cytoplasmic differentiation among populations was extremely high, with a mean significant *F*_ST_ value of 0.588 and of 0.743 for nuclear and cytoplasmic variation, respectively (Table 1 and Fig. S6).

### Population genetic structure of *B. vulgaris* subsp. *maritima* inferred from spatial multivariate ordination method

**Figure 4:**
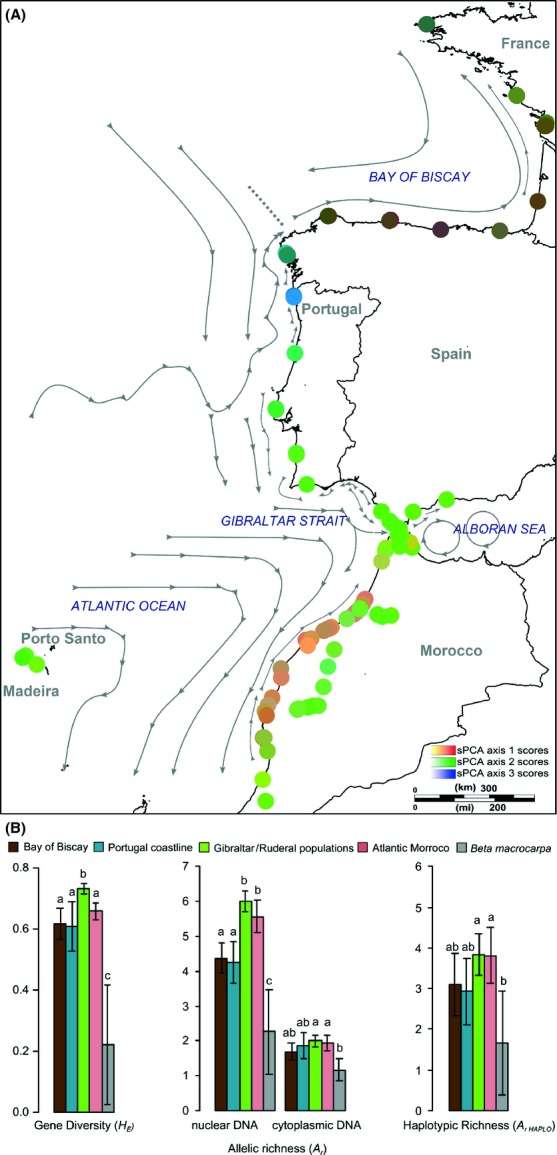
Genetic distinctiveness and mean levels of genetic diversity estimates for *B. vulgaris* subsp. *maritima* populations. (A) Genetic structure of *B. vulgaris* subsp. *maritima* populations revealed by a multivariate ordination method (sPCA) as described in Jombart et al. (2008). Color plot synthesizes the three first sPCA principal components at their respective spatial population coordinates. Each of the three axes was plotted on a red, green, and blue color scale, and the intensity of the color is proportional to the spatial principal component scores. Arrows indicate major surface currents redrawn from Koutsikopoulos and LeCann (1996), Millot (1999), Pelegrí et al. (2005), and Peliz et al. (2005). (B) Bar graph of the mean genetic diversity estimates (*H*_E_, *A*_r_*,* and *A*_r Haplo_) for each genetically distinct group of populations revealed by sPCA and for *B. macrocarpa* populations. Bars with different letters are significantly different at *P* < 0.05.

**Figure 5:**
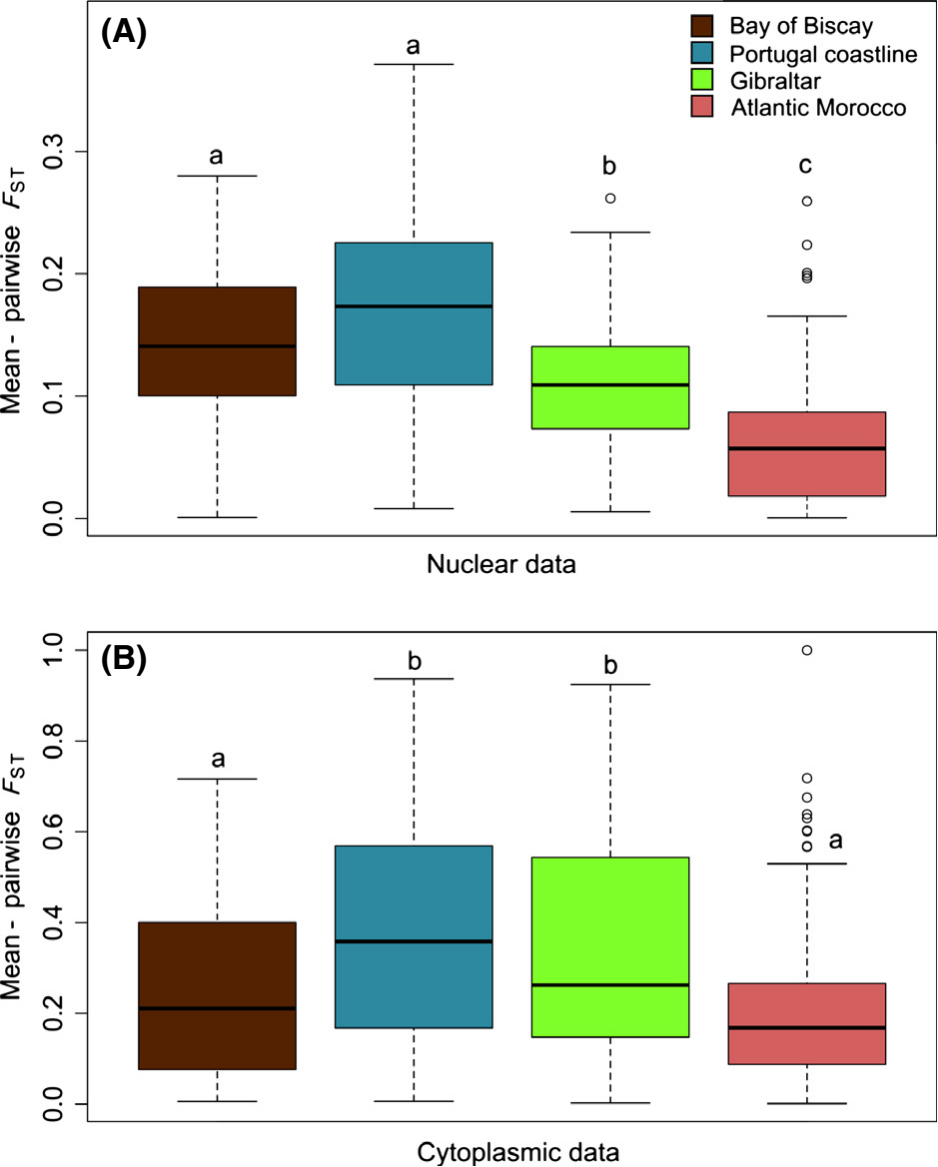
Box-plots of nuclear (A) and cytoplasmic (B) pairwise differentiation of populations (*F*_ST_) within genetic clusters depicted by sPCA. Box-plots indicate the median (horizontal line), the 25th and 75th percentiles (bottom and the top of the box), and the minimum/maximum values (vertical dashed lines). Plots with different letters are significantly different at *P* < 0.05.

The sPCA performed on the cytoplasmic data revealed no significant structure, either global (*P* = 0.681) or local (*P* = 0.184). When performing sPCA on nuclear data, a significant global structure was observed (*P* < 0.001), indicating that neighboring entities are genetically more similar than would be expected at random. The three first axes in the sPCA unambiguously captured all global spatial patterns (see Fig. S7). The resulting synthetic map defined four clearly different geographic entities: (1) the Bay of Biscay; (2) the Portuguese Atlantic coast, with a genetic cline between northern and southern populations; (3) the Strait of Gibraltar region on the Spanish and Moroccan sides; and (4) Moroccan southern Atlantic coast (Fig. 4A). Moreover, Moroccan inland ruderal populations clustered strongly with the group of populations located on the Moroccan side of the Strait of Gibraltar and southern Spain. Interestingly, populations located across the Strait of Gibraltar showed high autocorrelation values, suggesting no major genetic discontinuities in this area (Figs. 4A, S7). This analysis further traced back the evolutionary history of populations sampled on Madeira Island, which appear to originate from southern Portugal and Spain or northern Morocco near Gibraltar. Strongly concordant results were obtained with Bayesian clustering for the second modal value at *K* = 6 (see Fig. S2). Levels of genetic diversity for these genetically distinct groups of populations are depicted by both sPCA and Bayesian clustering in Figure 4B. Significant differences were found among groups for all variables (all at *P* < 10^−2^). Overall, the general trend was that populations located in Morocco and along the Strait of Gibraltar displayed the highest levels of cytoplasmic and nuclear genetic diversity. Figure 5 illustrates how pairwise genetic differentiation among populations differed between genetic clusters and shows a general increase in nuclear differentiation northward along the Atlantic coastline. Interestingly, cytoplasmic and nuclear data showed similar patterns with higher pairwise *F*_ST_ values observed along the Portuguese coastline (Fig. 5).

Lastly, including *B*. *macrocarpa* individuals in the sPCA clearly supported the high genetic distinctiveness between the two species observed with Bayesian analyses (see Fig. S8).

### Isolation-by-distance patterns

**Figure 6:**
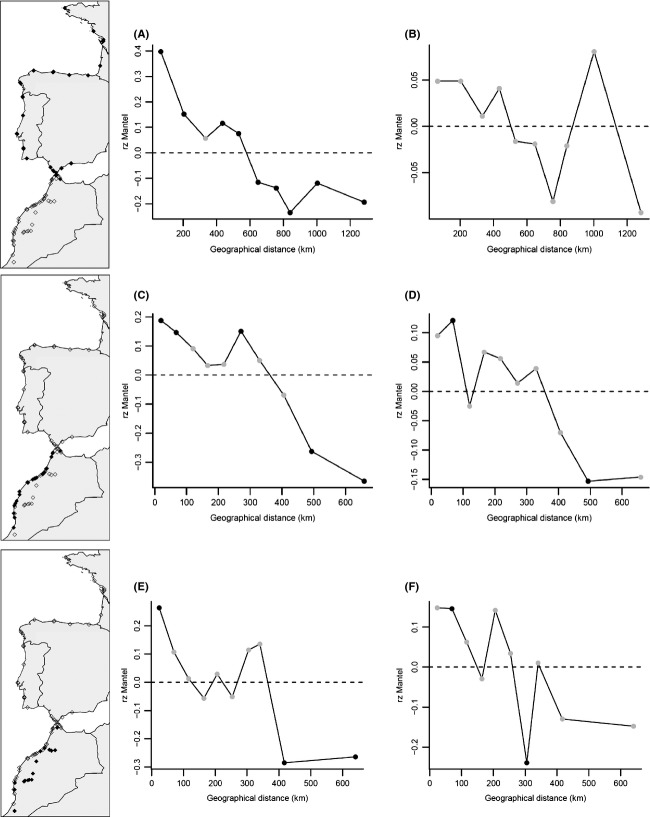
Mantel correlograms based on nuclear microsatellite (A, C, E) and mitochondrial minisatellite (B, D, F) data. Three groupings were investigated: northern populations (north of the Strait of Gibraltar) (A, B), Moroccan Atlantic populations (C, D), and inland ruderal populations (E, F). Black circles indicate a significant *rz* value at *P* < 0.05.

To design Mantel correlograms based on (1) previous results on population memberships and (2) a sufficient number of pairwise comparisons within distance classes to ensure robust statistical support, we only focused on three groups of *B. vulgaris* subsp. *maritima* populations that can be visualized in Figure 6. Using nuclear data, Mantel correlograms for northern populations up (north of the Strait of Gibraltar) revealed a clear continuous decrease in genetic similarity with increasing geographic distance (Fig. 6A). Focusing on Moroccan Atlantic populations, we observed moderately positive *rz* values stabilizing up to 280 km. From this distance, *rz* values substantially decreased (Fig. 6C). Regarding inland ruderal populations for nuclear data, only a positive short-distance autocorrelation and a long-distance genetic differentiation were observed (Fig. 6E). Finally, for cytoplasmic data*,* no significant spatial trends were observed: The correlograms showed fluctuations indicative of random genetic drift effects (Fig. 6B, D and F).

## Discussion

### Nuclear and cytoplasmic polymorphisms in *B*. *vulgaris* subsp. *maritima* and *B*. *macrocarpa*

One aim of this study was to elucidate spatial patterns of population genetic structure and draw indirect inferences on mating system based on genotypic structure, in two related species, *B. vulgaris* subsp. *maritima* and *B. macrocarpa*. One may wonder whether the modal *K* depicting three genetically distinct clusters clearly discriminated the two species. Nonetheless, we are very confident of the species assignation for the following reasons: (1) Concordant patterns of genetic distinctiveness were obtained using both sPCA and Bayesian clustering, (2) the hierarchical genetic differentiation was the highest between the two clusters of sea beets and the putative cluster composed of *Beta macrocarpa* individuals, and (3) contrasting patterns of genetic differentiation among populations and genotypic structure within populations were found between the two clusters of sea beets and the cluster composed of *Beta macrocarpa* individuals, the observed patterns being concordant with what is known about the mating system in these two species (Letschert 1993; Bruun et al. 1995) and more generally in plant species displaying different reproductive traits (Hamrick and Godt 1996). Therefore, the third cluster we depicted is clearly unlikely to correspond to an additional cluster of sea beets that would be characterized by complete autogamy. Overall, significant cytoplasmic and nuclear genetic differentiation was found among 85 *B. vulgaris* subsp. *maritima* and 8 *B. macrocarpa* populations. Mitochondrial DNA diversity revealed considerably more population structuring than that found for nuclear DNA, as shown by the strong asymmetry in *F*_ST_ values. This finding can be explained by (1) the larger effective population size for nuclear genes as compared to organellar genes, indicating a stronger effect of genetic drift on cytoplasmic DNA, and (2) higher gene flow for nuclear DNA, which occurs via seed and pollen dispersal, than for cytoplasmic DNA, which takes place via seed movements only (Ennos 1994). In *B*. *vulgaris* subsp. *maritima*, the amount of genetic differentiation was fairly high compared with those found in previous studies involving a metapopulation structure at microgeographic scale less than a few hundred meters (De Cauwer et al. 2010) or involving a regional structure (Fievet et al. 2007). This is in accordance with the fact that genetic differentiation generally increases with geographic distance (reviewed in Petit et al. 2005). However, the contrast between nuclear and cytoplasmic differentiation observed over a wide species-range scale appeared reduced compared with those found in studies performed at finer geographic scales. The large differences between seed and pollen dispersal may indeed be more difficult to detect due to the greater action of genetic drift and mutation, with differentiation being random at large geographic scales (Hutchison and Templeton 1999; Fievet et al. 2007). Furthermore, founder events involving rare propagules can undergo strong genetic drift, as suggested by the lack of large-scale structuring for cytoplasmic diversity (Whitlock and McCauley 1990). This type of metapopulation dynamics is plausible given that sea beets occur in coastal environments where frequent disturbance may lead to extinctions and recolonizations (De Cauwer et al. 2012). This explanation is consistent with hierarchical *F*-statistics analyses, revealing that most differentiation occurs among populations within geographic groups.

*Beta macrocarpa* populations exhibited very low genetic variation, large heterozygote deficiency, and a high degree of genetic differentiation compared with *B*. *vulgaris* subsp. *maritima* populations. Herbaceous annuals with seed dispersal by gravity and mixed-mating or pure selfing mating systems usually exhibit higher *F*_IS_ values and stronger genetic differentiation than outcrossing species (Hamrick and Godt 1996; Duminil et al. 2009). Accordingly, this suggested that selfing could be the main reproductive mode of *B. macrocarpa*. This is therefore consistent with a self-compatible mating system documented by Buttler (1977), Lange and Debock (1989), Letschert (1993), and Bruun et al. (1995). Interestingly, two populations located in Santo Porto showed mean single-locus *F*_IS_ values and multilocus genotypes indicative of individual fixed heterozygosity, as also documented by Letschert (1993) on tetraploid (*2n* = 36) accessions from Canary Islands. Along with the recent discovery of diploid and tetraploid forms occurring in sympatry in south Portugal (Castro et al. 2013), our findings also suggest the mixing of diploid and tetraploid variants in *B. macrocarpa* located in Madeira Islands. Inbreeding depression is weaker in tetraploids, which may be a determining factor in the evolution of polyploidy (Husband and Schemske 1997). Indeed, polyploidy appears to be especially prevalent in self-fertilizing annual colonizing plants, and it is often argued that heterozygosity is a key element in their colonizing ability (reviewed in Soltis and Soltis 2000). In *B. macrocarpa*, tetraploidy may thus be adaptively significant because this species often occurs in disturbed environments. Nonetheless, further experiments involving chromosome counting and flow cytometry are necessary to accurately document the occurrence of diploid and tetraploid variants in sympatry.

### *B. vulgaris* subsp. *maritima*: do inland populations have peculiar evolutionary histories?

To date, there are only few examples of typical coastal species that also occur inland – albeit sparsely – especially halophytic species. Comes and Abbott (1998) observed lower genetic diversity in inland populations of *Senecio gallicus* and suggested that they originated from coastal populations. However, lower genetic diversity for inland populations is not a common feature (e.g., Krüger et al. 2002; Prinz et al. 2009). In our study, Moroccan inland ruderal populations exhibited higher levels of genetic diversity than coastal populations. In addition, no clear isolation-by-distance (IBD) pattern was detected, contrary to spatial genetic structure commonly observed in coastal populations (Fievet et al. 2007). Only positive autocorrelation of short-distance classes was observed, likely reflecting genetic clustering and family structuring caused by limited short-range dispersal of the multigerm seedball (De Cauwer et al. 2012). The lack of IBD patterns at larger spatial scales can be attributed – at least partially – by recent spreads along with human-caused habitat disturbances, which blurs the spatial patterns of genetic relatedness by modifying the movement of dispersal vectors and significantly changing the number and distribution of recruitment sites (González-Martínez et al. 2010). Pollen flow may also be very localized and constrained by inland topography, as opposed to the propitious pollen dispersal in open habitats such as shorelines (Arnaud et al. 2009; De Cauwer et al. 2010).

Finally, Moroccan inland ruderal populations clustered with coastal populations in the Gibraltar region, a result consistent with both sPCA and Bayesian analyses (Figs. 3, S2). Again, these results could highlight a recent expansion of *B. vulgaris* subsp. *maritima* from the Mediterranean coast, possibly mediated by human-induced long-dispersal events as suggested by Desplanque et al. (1999) and Arnaud et al. (2009) for southern European inland wild beet populations. Ruderal habitats are common elements in the Mediterranean landscape due to land-use changes (Naveh and Vernet 1991). Therefore, opportunistic growth and rapid reproduction in unstable habitats appear to have played an important role in the inland expansion of weedy beet relatives (Arnaud et al. 2011). Inland ruderal Moroccan populations further appeared to have admixed origin from coastal European and Moroccan lineages. The admixture of different lineages and subsequent multiple introductions in a new habitat can provide large amounts of genetic diversity, novel sources of genetic combinations in plant species complexes, and may thus offer opportunities to intensify the adaptability of populations to a new niche on microevolutionary timescales (Ellstrand and Schierenbeck 2000; Barrett et al. 2008). A striking example of recurring pattern of adaptive evolution in *B. vulgaris* is the evolution of invasive weed beets from crop–wild hybrids that entails numerous changes in life history, morphology, and ecology. It has indeed been shown that the invasiveness of weedy lineages arises through the introgression of a wild genetic background into the crop gene pool (Arnaud et al. 2010). This wild genetic background comes from inland ruderal beets found in disturbed habitats in southern France, which have very similar ecology as inland Moroccan beets (Desplanque et al. 1999; Arnaud et al. 2009). Indeed, in southern France, these noncoastal wild beet populations have colonized disturbed, human-influenced habitats, thanks to specific life-history traits that enhance colonizing abilities: the timing of flowering, the seed dormancy associated with a long-lived seed bank, and the breakdown of self-incompatibility (Desplanque et al. 1999; Hautekèete et al. 2002; Arnaud et al. 2011). These traits may also be crucial in inland Moroccan populations sharing very similar ecological conditions, that is, unpredictable and man-made environments at low latitude. Overall, in such disturbed-like environments, the combination of reproductive traits and life-history traits due to admixture of different lineages may facilitate the adaptive evolution of inland beet lineages and could facilitate their colonization capabilities, as they do for weedy invasive lineages found in cultivated fields (Arnaud et al. 2010). In the future, this could be of particular concern in the major areas of cultivation of sugar beets in central west Morocco.

Lastly, founder effects during colonization are likely to involve ample mixing of seeds due to pastoralism and grazing activities along field edges (Pereira et al. 2009). Multiple sources of introduction and/or different successive independent colonization events from the Mediterranean coast may therefore explain the admixture, the lack of IBD, and the higher levels of genetic diversity found in inland populations. Newly developed ABC methods could be useful to elucidate the origins of inland populations (e.g., Lombaert et al. 2011). However, our data set does not conform to current ABC computations in two aspects. First, we dealt with a very large number of populations not amenable to current ABC analyses without size reduction. Second, the among-population genetic structure observed in each compartment (coastal and ruderal) is high, which can lead to the inference of erroneous scenarios. Potential solutions exist to circumvent these difficulties, by modeling unsampled populations and/or using different sets of sampled populations (E. Lombaert, pers. comm.). Testing the appropriateness of these different solutions is, however, beyond the scope of this paper. As such, we acknowledge that concluding on a particular evolutionary history of inland populations remains speculative as long as these scenarios have not been thoroughly tested.

### Phylogeographic patterns in *B. vulgaris* subsp. *maritima*

Understanding the underlying mechanisms of range shifts and the determinants of population establishment are fundamental questions in evolutionary biology and ecological genetics and more importantly in the context of changing climate. The patterns we document in *B. vulgaris* subsp. *maritima* provide clues for understanding how life-history and major geographic features interact to shape the distribution of genetic diversity.

#### Does the Strait of Gibraltar impede gene flow?

The Mediterranean-Atlantic transition has been reported to be an efficient barrier to the expansion of many coastal plants and marine species along the coast (Kadereit et al. 2005; Westberg and Kadereit 2009). Nonetheless, this barrier effect remains uncertain in *B. vulgaris* subsp. *maritima* because we found a clear genetic similarity among populations located on both sides of the Strait of Gibraltar. This lack of genetic structuring suggested that there is no major biogeographic barrier in this area, which can be explained by: i) colonization before barrier formation and/or ii) effective dispersal after barrier formation. During the last glacial maxima, the relative sea level dropped 120 m relative to its present level and the distance between the two continents was thereby reduced to 10 km (Yokoyama et al. 2000). In addition to the proximity between shores, small islands and islets emerged and may have provided pathways of migration through a stepping-stone-like network between the two continents (Collina-Girard 2001). Alternatively, it may involve long-distance seed dispersal events favored by sea currents and efficient pollen flow. As found in *B. vulgaris* subsp. *maritima*, genetic continuities on both sides of the strait have been reported for several herbaceous plant species characterized by ruderal life-history traits (e.g., Caujapé-Castells and Jansen 2003; Ortiz et al. 2007). These findings are in sharp contrast with other studies that examined long-lived plants such as trees, which tend to show genetic breaks associated with the strait (e.g., Lumaret et al. 2002). Overall, it appears that the Strait of Gibraltar filtered plant migration according to their dispersal and life-history traits (Lavergne et al. 2013).

#### Influence of marine currents

Seed dispersal is a major determinant of the spatial genetic structure of populations (Ennos 1994). In most cases, *B. vulgaris* subsp. *maritima* seeds are dispersed by gravity in the vicinity of the mother plant (De Cauwer et al. 2012). However, secondary seed dispersal through water transport has been suggested because buoyant seeds can be carried away during the highest tides and subsequently drifted in marine waters (Fievet et al. 2007). Interestingly, the main phylogeographic breaks depicted by both sPCA and Bayesian clustering analysis matched the main surface current discontinuities (see Fig. 3). The sharp genetic change observed in Galicia, at the western tip of the Iberian Peninsula, coincides with a water-mass front located off Cape Finisterre and has been reported as a major dispersal barrier for several marine organisms such as algae and marine snails with planktonic larvae being released in seawater (Perez et al. 1993; Alberto et al. 1999; Piñeira et al. 2008). Furthermore, an additional biogeographic barrier has been identified in Portugal at 41°N where the eastern North Atlantic surface currents bifurcate (e.g., Diekmann et al. 2005; Coyer et al. 2011). The resulting south-flowing Portuguese current and the northeast-flowing Portuguese counter-current may thus account for the genetic cline and increased levels of pairwise genetic differentiation observed in *B. vulgaris* subsp. *maritima* populations along the Portuguese Atlantic coast (see Martins et al. 2002). Finally, the substantial genetic distinctiveness between southern Moroccan Atlantic populations and populations found in the Strait of Gibraltar region on the Spanish and Moroccan sides corresponds to a physical discontinuity of marine currents, as shown in Figure 3. Thus, large-scale oceanographic features along the coastline may affect seed dispersal and population connectivity in wild sea beet, leading to genetically differentiated lineages similar to what can be observed in marine organisms (e.g., Alberto et al. 1999; Coyer et al. 2011). Lastly, drifting through seawater can facilitate seed dispersal, which is very limited along the terrestrial shoreline. Indeed, sea beet seeds have no particular dispersal mechanism except a gravity-driven pattern of dispersal. As a consequence, most seeds are dispersed at short distances near the mother plants in terrestrial areas (Arnaud et al. 2011; De Cauwer et al. 2012). A high genetic differentiation among a mosaic of genetically distinct demes is thus found within populations, with striking genetic discontinuities taking place within a few tens of meters (De Cauwer et al. 2012). Moreover, although being a wind-pollinated species, pollen flow is spatially localized and constrained by landscape topography (Fievet et al. 2007; De Cauwer et al. 2010, 2012). This explains the strong IBD pattern found for coastal populations at nuclear microsatellite loci.

#### Geographic trends in the levels of genetic diversity and structure: footprints of postglacial recolonization?

It seems unlikely that *B. vulgaris* subsp. *maritima* survived the Würm glaciation: Due to ice and permafrost, it would not have been able to maintain a continuous distribution along large parts of the Atlantic coast of Europe. Today, the northern range limit of *B. vulgaris* subsp. *maritima* is situated at approximately 58°N near the 8°C January and 14°C July isotherms. Therefore, populations probably had to retreat to at least northern Portugal, where the 14°C July isotherm would have been located during the last glacial maximum (see Fig. S9). In accordance, *Eryngium maritimum*, a coastal species that shares the same habitats, temperature requirements, and distribution range as *B. vulgaris* subsp. *maritima*, was estimated to have retreated to as far south as North Africa (Clausing et al. 2000). Because rare alleles are expected to be lost with bottlenecks occurring during subsequent recolonization and range expansion, patterns of central-marginal and latitudinal gradients in genetic diversity can point to refuge sources (Austerlitz et al. 1997). Consistently, a clear and significant decrease in allelic richness and gene diversity with increasing latitude was observed from the Gibraltar region, which exhibited a high level of genetic diversity and many private alleles at nuclear loci. This pattern of “southern richness” is characteristic of temperate terrestrial taxa that persisted through past glacial conditions in southern refugia (Hampe and Petit 2005). This is also indicative of a central-marginal trend in decreasing diversity (see Guo 2012) and may corroborate the Gibraltar region as a historical refuge for *B. vulgaris* subsp. *maritima*. Indeed, the Gibraltar region is considered as a buffer zone that was climatically stable during geological times due to its latitude and the influence of the Atlantic Ocean and the Mediterranean Sea (Rodríguez-Sánchez et al. 2008). However, we cannot rule out secondary contacts between two divergent lineages originating from the eastern Mediterranean and southern Moroccan coast. The high levels of admixture found in northern Moroccan coastal populations are consistent with the occurrence of genetically diverse suture zones similar to glacial refugia (Sakaguchi et al. 2011). The high genetic diversity observed in this area may also suggest past abrupt increase in migration among populations. Indeed, such transient dynamics has been shown to leave genetic diversity peaks that can be conserved over thousands of generations, as theoretically demonstrated by Alcala et al. (2013).

Subsequently, the significant decrease in nuclear gene diversity and allelic richness northward from the Strait of Gibraltar region can be attributed to a series of founder effects during range expansion along the shoreline. In this type of narrow expanding population front, drift effects may be accentuated by growth of genetically uniform populations ahead of the main colonization front that would inhibit subsequent gene flow (Waters et al. 2013). The strong IBD pattern observed for nuclear diversity also points to a scenario analogous to the propagule-pool model developed for landscapes with extinctions-recolonization dynamics, that is, founders are drawn from a limited number of source populations consequently reducing population genetic diversity and increasing the genetic differentiation among populations (Wade and McCauley 1988; Whitlock and McCauley 1990; Giles and Goudet 1997; Haag et al. 2005). Indeed, in Europe, sea beets are generally patchily located along the sea shore, where high tides, seasonal storms, and anthropogenic disturbances cause frequent extinctions of populations.

In contrast, we did not find any IBD patterns for cytoplasmic variation, nor any evidence for decreasing levels of cytoplasmic diversity or increase in differentiation among newly established northern Atlantic populations following the postglacial recolonization. A migrant-pool model – where colonization occurs from multiple source populations (Slatkin 1977) – would be more likely to fit the spatial patterns of cytoplasmic variation. At first glance, these results appear puzzling as several sea beet seeds are aggregated in a single fruit, which provides the opportunity for kin-structured founder effects (Whitlock and McCauley 1990; De Cauwer et al. 2012). Similar contrasting results between nuclear and cytoplasmic diversity have been observed in plants (e.g., García-Verdugo et al. 2010) and could be interpreted by the occurrence of multiple stochastic long-distance seed migration mediated by major marine currents, as suggested in our study by the depicted phylogeographic breaks that matched some major marine current discontinuities. Seed dispersal by marine currents may indeed possibly compensate the founder effects by gene mixing despite a narrow coastline habitat (Fayard et al. 2009).

## Conclusion

Nearly complete autogamy was verified in *B. macrocarpa*. Gradients in allelic richness and gene diversity in *B*. *v*. subsp. *maritima* suggested a scenario of postglacial recolonization from the Mediterranean-Atlantic region and supported the status of southern Iberia and Morocco as a long-term refuge as documented for a wide range of organisms. Further, specific life-history traits along with the admixture from genetically differentiated lineages are likely to increase invasiveness for ruderal lineages in anthropogenically disturbed habitats. Our results also indicate that environmental factors, such as marine currents, may play a role in shaping genetic diversity in coastal plant species concordantly of what can be observed in marine organisms releasing larvae in water flows. Range expansions do not always have straightforward consequences: in our case study, long-distance seed dispersal through marine currents may drive to a mixing of cytoplasmic diversity and buffer the northward decrease in nuclear allelic richness observed for edge populations recently established. This challenges the usual view that populations located at the edge of a species' geographic distribution are genetically depauperate.

## Data Accessibility

Coordinates of sample locations, cytoplasmic, and nuclear genetic data are available in DRYAD: doi: 10.5061/dryad.m1n38.

## Appendix

Population localities, label, geographic coordinates, sample sizes, and summary of genetic diversity for nuclear polymorphism at eight microsatellite loci and for cytoplasmic polymorphism at four minisatellite loci among 93 wild *Beta vulgaris* subsp. *maritima* (Mari) and *Beta macrocarpa* (Macro) populations. *B. macrocarpa* populations are shaded in gray. *H*_E_, gene diversity within populations; *A*_r_, allelic richness; *A*_r_*P*, private allelic richness; *A*_*n*_, mean number of alleles; *F*_IS_, the intrapopulation fixation index (**P* < 0.0001); *Nb Haplo*, number of haplotypes; *A*_r__Haplo_, haplotypic richness. *A*_r_ is the allelic richness corrected to *n* = 8 using the rarefaction method of El Mousadik and Petit (1996) implemented in FSTAT version 2.9.3.2.

**Table:**

							Nuclear					Cytoplasmic		
									
Region	Label	Sampling locality	Latitude	Longitude	*n*	Species	*H*_E_	*A*_r_	*A*_r_*P*	*A*_*n*_	*F*_IS_	*Nb Haplo*	*A*_r Haplo_	*A*_r_
France	1	Bretagne Aber Ildut	48.46672	−4.75677	15	Mari	0.537	4.072	0.001	5.000	0.158	2	2.000	1.500
	2	Bretagne Pors Gored	48.46492	−4.76597	20	Mari	0.516	3.711	0.000	4.500	0.079	3	2.870	1.913
	3	St Gilles-Croix-de-Vie	46.70918	−1.98033	11	Mari	0.715	4.790	0.091	5.125	−0.048	5	4.803	2.244
	4	St Gilles-Croix-de-Vie	46.69930	−1.97313	11	Mari	0.660	4.142	0.053	4.375	−0.034	1	1.000	1.000
	5	Yves	46.03008	−1.05638	20	Mari	0.761	5.938	0.009	8.000	0.085	6	4.813	2.008
	6	Fouras	45.95538	−1.06833	20	Mari	0.692	5.350	0.003	7.875	−0.011	9	6.882	2.805
	7	Île Madame	45.95347	−1.11120	20	Mari	0.729	5.384	0.010	7.250	0.045	5	3.929	1.954
	8	Aquitaine Contis plage	44.08869	−1.32183	14	Mari	0.604	4.158	0.004	4.750	0.069	3	2.976	1.498
Iberian peninsula	9	Lekeitio	43.36063	−2.49835	24	Mari	0.637	4.629	0.006	6.375	0.149	4	3.375	1.619
	10	Oyembre	43.38925	−4.32932	39	Mari	0.578	4.444	0.001	8.250	0.148	4	2.892	1.609
	11	Verdicio	43.62828	−5.87637	26	Mari	0.556	3.668	0.001	5.250	0.082	2	1.994	1.249
	12	Verdicio	43.62478	−5.87912	15	Mari	0.386	2.430	0.003	2.750	0.118	2	1.963	1.246
	13	Xago	43.60868	−5.91265	15	Mari	0.643	4.512	0.002	5.500	0.129	2	2.000	1.250
	14	Celtigos	43.72675	−7.79440	16	Mari	0.619	4.126	0.001	4.750	0.056	3	2.516	1.613
	15	Celtigos	43.72542	−7.79452	15	Mari	0.583	3.979	0.033	4.750	0.139	3	2.790	1.674
	16	Espasante	43.72255	−7.81105	20	Mari	0.678	4.763	0.006	6.625	−0.033	3	2.646	1.926
	17	Texueiro	42.80988	−9.11333	18	Mari	0.517	3.974	0.067	5.000	0.015	2	1.698	1.189
	18	San Francisco	42.75742	−9.07678	14	Mari	0.598	4.327	0.008	6.125	0.111*	3	2.853	2.198
	19	San Francisco	42.76038	−9.06135	21	Mari	0.628	4.591	0.000	5.500	−0.081	4	3.861	2.250
	20	Paço	41.75697	−8.87668	18	Mari	0.543	3.290	0.003	3.750	−0.137*	2	1.996	1.250
	21	Areosa	41.72123	−8.87067	17	Mari	0.432	3.104	0.003	3.750	−0.072	3	2.758	2.347
	22	Areosa	41.72217	−8.86568	17	Mari	0.407	2.800	0.050	3.500	−0.04	2	2.000	1.500
	23	Praia da Tocha	40.33647	−8.83982	13	Mari	0.734	5.030	0.000	6.000	0.069	4	3.723	2.186
	24	Ericeira	38.96197	−9.41917	10	Mari	0.675	4.184	0.000	4.750	0.053	5	4.833	2.676
	25	Ericeira	38.96460	−9.43267	15	Mari	0.735	5.097	0.000	5.375	0.064	3	2.968	1.499
	26	Lizandro	38.94468	−9.41478	15	Mari	0.713	5.382	0.000	7.000	−0.028	5	4.509	2.415
	27	Samoqueira	37.86950	−8.79295	15	Mari	0.715	4.988	0.025	6.125	−0.095*	1	1.000	1.212
	28	Porto Covo	37.84960	−8.79315	15	Mari	0.728	6.045	0.067	7.875	0.01	6	5.450	2.815
	29	Pessegueiro	37.82962	−8.79122	10	Mari	0.756	6.400	0.009	6.875	0.103*	5	5.000	2.995
	30	Carvoeiro	37.09582	−8.47155	26	Mari	0.684	5.511	0.074	8.500	0.161*	4	3.919	1.988
	31	Chiclana de la Frontera	36.42903	−6.15942	15	Mari	0.821	7.081	0.099	9.250	0.107*	5	4.576	2.348
	32	Barbate	36.19350	−5.90813	21	Mari	0.748	5.940	0.010	8.750	−0.02	2	1.646	1.510
	33	Zahara	36.13983	−5.85178	15	Mari	0.752	6.394	0.046	8.250	0.047	5	4.713	1.953
	34	Tarifa	36.0095	−5.60633	22	Mari	0.833	7.329	0.097	11.500	−0.03	6	5.298	2.844
	35	Estepona	36.41867	−5.17850	32	Mari	0.699	5.042	0.061	7.625	0.071*	2	2.000	1.250
	36	Almayate	36.72283	−4.14383	32	Mari	0.816	6.325	0.005	9.625	0.071*	4	3.775	1.961
Morocco	37	Cap Mazari	35.53830	−5.21467	24	Mari	0.744	6.099	0.076	9.500	0.019	4	3.120	1.864
	38	Cabo Negro	35.66600	−5.28470	24	Mari	0.653	4.824	0.049	6.875	0.198*	3	2.866	1.476
	39	Ksar-as-Seghir	35.84418	−5.55490	24	Mari	0.753	5.651	0.077	7.750	−0.016	2	2.000	1.250
	40	Ksar-as-Seghir	35.82791	−5.65704	33	Mari	0.692	4.421	0.226	6.500	−0.033	3	1.858	1.602
	41	Cap Malabata	35.79852	−5.75094	26	Macro	0.045	1.234	0.000	1.250	1.000*	1	1.000	1.000
	42	North of Asilah	35.53147	−6.00637	25	Mari	0.724	6.146	0.017	9.000	0.012	3	2.786	1.460
	43	Asilah	35.47471	−6.02639	15	Mari	0.683	5.332	0.037	6.875	0.127*	4	3.801	2.158
	44	Asilah	35.46424	−6.04214	19	Mari	0.790	5.841	0.279	7.375	0.01	1	1.000	1.000
	45	Larache	35.19856	−6.15395	34	Mari	0.713	5.431	0.120	8.750	0.006	4	3.254	1.585
	46	Mehdiya harbor	34.26198	−6.66486	10	Mari	0.573	3.609	0.004	3.625	−0.025	1	1.000	1.000
	47	Mehdiya beach	34.24847	−6.68020	15	Mari	0.522	3.925	0.000	4.750	0.043	3	2.963	1.741
	48	Bouknadel	34.14674	−6.73757	31	Mari	0.636	5.399	0.008	8.125	0.096	5	3.652	1.699
	49	Rabat	34.03328	−6.83532	24	Mari	0.679	5.541	0.090	8.500	0.041	4	3.914	2.234
	50	Rabat	33.95541	−6.92240	14	Mari	0.692	6.664	0.027	8.625	−0.021	7	6.425	3.153
	51	Temara	33.92765	−6.96061	17	Mari	0.701	6.361	0.069	8.750	0.082*	5	4.168	2.035
	52	Skhirat beach	33.89583	−7.00148	12	Mari	0.643	5.697	0.091	6.500	0.087	3	2.933	1.737
	53	Casablanca	33.57103	−7.70927	15	Mari	0.692	6.305	0.032	8.125	0.036	5	4.372	1.920
	54	Casablanca	33.53502	−7.78873	16	Mari	0.654	6.102	0.090	8.250	0.104*	4	3.515	1.863
	55	Tamaris Beach	33.52242	−7.84612	17	Mari	0.684	6.064	0.010	8.625	0.054	3	2.991	1.499
	56	Rit Reima	33.47979	−7.94681	13	Mari	0.702	6.361	0.159	7.750	0.083*	4	3.723	1.956
	57	El Jadida	33.25606	−8.50058	23	Mari	0.653	5.782	0.032	8.750	0.019	5	4.096	2.055
	58	Sidi Moussa	32.54697	−9.27294	15	Mari	0.617	5.413	0.060	6.875	−0.013	3	2.952	1.992
	59	Safi	32.32353	−9.26135	25	Mari	0.660	5.797	0.003	8.250	0.09	6	5.590	2.413
	60	El Mehattat	31.82972	−9.54357	24	Mari	0.700	6.497	0.030	9.500	0.018	4	3.802	1.996
	61	Moulay-Bouzerktoun	31.64687	−9.66897	21	Mari	0.689	6.279	0.053	9.500	0.069*	7	5.951	2.462
	62	Essaouira	31.49162	−9.76452	26	Mari	0.716	6.665	0.081	11.250	0.122*	6	5.396	2.126
	63	Immesouanne Beach	30.84024	−9.82156	20	Mari	0.524	3.337	0.021	4.250	−0.113	1	1.000	1.000
	64	Immesouanne Beach	30.83945	−9.82058	20	Mari	0.647	4.310	0.004	5.125	−0.067*	4	3.993	2.150
	65	Tarhazoute	30.56927	−9.74449	18	Macro	0.100	1.735	0.000	1.750	0.926*	1	1.000	1.000
	66	North of Tarhazoute	30.56915	−9.74450	30	Macro	0.062	1.470	0.235	1.750	0.723*	1	1.000	1.000
	67	Imourane Beach	30.50862	−9.68645	4	Mari	0.766	–	–	4.375	−0.184*	–	–	–
	68	Aglou Beach	29.80457	−9.83363	20	Mari	0.687	5.217	0.101	7.375	−0.146	–	–	–
	A	Tétouan	35.56475	−5.59577	24	Mari	0.766	5.808	0.090	8.375	0.109*	4	3.374	1.772
	B	Khemisset	33.86032	−5.86353	20	Mari	0.727	5.998	0.019	8.375	−0.049	7	5.586	2.792
	B	Khemisset	33.86032	−5.86353	4	Macro	–	–	–	2.875	0.657*	1	–	–
	C	Tiflét	33.83300	−6.13092	24	Mari	0.762	6.843	0.163	11.500	0.071*	5	4.458	2.136
	D	Tiflét	33.88387	−6.2798	24	Mari	0.673	5.289	0.000	7.250	0.080*	3	2.245	1.547
	E	Rabat Center	34.01871	−6.82016	13	Mari	0.726	6.351	0.120	7.750	0.192*	6	5.570	2.455
	F	Bouznika	33.77795	−7.23266	21	Mari	0.729	6.489	0.031	9.750	0.118*	2	1.998	1.476
	G	Azemmour	33.28874	−8.33113	19	Mari	0.691	5.734	0.021	8.000	0.076*	5	4.316	1.864
	H	Sebt-des-Oulad-Hassine	33.11637	−8.44159	2	Mari	–	–	–	–	–	–	–	–
	H	Sebt-des-Oulad-Hassine	33.11637	−8.44159	6	Macro	0	–	–	1.000	–	1	–	–
	I	Settat	33.01755	−7.62048	33	Mari	0.787	6.826	0.021	11.750	0.151*	9	6.15	2.880
	J	El Guelb	32.59028	−7.81497	4	Mari	0.620	–	–	3.375	−0.109	1	–	–
	J	El Guelb	32.59028	−7.81497	37	Macro	0.073	1.770	0.062	2.375	0.716*	1	1.000	1.000
	K	Benguerir	32.09525	−7.95204	15	Mari	0.722	6.887	0.182	9.250	−0.022	7	6.466	2.452
	L	Marrakech	31.62862	−8.11320	18	Mari	0.806	7.253	0.087	10.375	0.070*	5	4.954	2.187
	M	Zoudia	31.62891	−8.26613	19	Mari	0.744	6.629	0.164	9.750	0.115*	7	6.051	2.611
	N	Oudaya	31.59095	−8.43793	11	Mari	0.764	6.294	0.010	6.625	0.091	4	3.993	2.250
	O	Chichaoua East	31.55745	−8.72626	14	Mari	0.767	6.654	0.072	8.625	−0.013	4	3.933	2.213
	P	Chichaoua Center	31.54526	−8.75929	18	Mari	0.779	7.559	0.100	11.500	0.101*	8	6.890	2.671
	Q	Chichaoua	31.53588	−8.76460	29	Mari	0.748	7.170	0.090	12.625	0.081*	6	4.696	2.231
	R	South of Essaouira	31.39083	−9.71322	14	Mari	0.634	4.357	0.125	5.000	−0.029	3	2.823	1.470
	S	Immesouanne	30.84051	−9.78695	19	Mari	0.754	6.664	0.021	9.250	0.048	5	4.559	2.597
	T	North of Guelmin	29.26903	−9.73838	16	Mari	0.739	6.406	0.355	8.750	0.006	4	3.516	2.159
Santo Porto Island	69	Serra de Dentra	33.08833	−16.3102	16	Macro	0.535	3.375	0.001	3.375	−0.461*	2	2.000	1.250
	70	Adega das Levadas	33.03647	−16.37742	17	Macro	0.528	4.051	0.000	4.125	0.234*	4	3.996	1.749
Madeira Island	71	Porto Moniz	32.86643	−17.1663	21	Mari	0.605	3.656	0.017	4.375	0.115*	1	1.000	1.000
	72	Ponta do Pargo	32.81427	−17.26288	24	Mari	0.702	4.121	0.000	4.750	−0.01	2	1.815	1.215
	73	Caniço de Baixa	32.64597	−16.82443	18	Mari	0.745	5.242	0.006	7.000	0.194*	5	4.596	1.924
Mean multilocus estimates						Mari	0.678	5.367	0.052	7.239	0.050*	3.965	3.573	1.915
						Macro	0.223	2.272	0.050	2.313	0.769*	1.500	1.666	1.167

## Supporting Information

**Figure S1.** Assignment results from Bayesian clustering following Pritchard et al. (2000) performed on (A) *B. vulgaris* subsp. *maritima* and *B. macrocarpa* individuals (B) *B. vulgaris* subsp. *maritima* individuals only; and, (C) following Durand et al. (2009) on both species. STRUCTURE analyses (A,B): mean (±SD) probabilities of the data Ln Pr(X|*K*) over 15 replicated runs plotted as a function of the putative number of clusters *K* (blue dots) and the standardized second-order rate of change of Ln Pr(X|*K*), Δ*K*, as a function of *K* (red dots). TESS analyses (C): average Deviation Index Criterion (DIC) over 20 replicated runs plotted against *K*.

**Figure S2.** Assignment probabilities of membership of (A) *B. vulgaris* subsp. *maritima* individuals into the five inferred clusters for the second modal value, and (B) *B. vulgaris* subsp. *maritima and B. macrocarpa* individuals into the six inferred clusters for the second modal value. Each individual is represented by a thin horizontal line (*y* axis) partitioned into coloured segments that represented the individual's estimated membership coefficients (*x* axis) (C) Map of mean population membership probabilities for the six clusters.

**Figure S3.** Population genetic structure of *B. vulgaris* subsp. *maritima* inferred from TESS analyses assuming *K* = 5. The individual estimates membership coefficients for each cluster are shown for 20 independent runs.

**Figure S4.** Geographical distribution of minisatellites haplotypes within populations.

**Figure S5.** Linear regressions of genetic diversity indices based on nuclear (*A*_r_*, H*_E_*, A*_r_*P*) and based on cytoplasmic (*A*_r_) polymorphism with respect to latitude and coastline distance for each locus. Regressions were performed on (A) northern populations (labelled 1 to 40 in Fig. 1), and (B) Moroccan populations at lower latitudes (labelled 43 to *t*).

**Figure S6.** Matrices of population-pairwise differentiation values (*F*_ST_) of *B. vulgaris* subsp. *maritima* and *B. macrocarpa* individuals represented for (A) nuclear and (B) cytoplasmic data.

**Figure S7.** Individual representation of the three first global axes of the spatial principal component analysis (sPCA) performed on *B. vulgaris* subsp. *maritima* populations. According to the first three global eigenvalues, First (A), second (B), and third (C) sPCA scores, are represented on each plot as squares whose size is proportional to the value of the score, so that the maximum differentiation is between large white squares and large black squares.

**Figure S8.** Individual representation of the three first global axes of the spatial principal component analysis (sPCA) performed on *B. vulgaris* subsp. *maritima* and *Beta macrocarpa* populations.

**Figure S9.** Modern (12°C, 14°C and 16°C mean July, northern Europe) and reconstructed (June/July/August isotherms, southern Europe) Last Glacial Maximum (LGM) isotherms from Kadereit et al. 2005;. Bold lines indicate the ice shield during the LGM and dotted lines indicate the coastline during the LGM.

**Table S1.** Mantel tests carried out on genetic distance matrices based on nuclear and cytoplasmic data for *B. vulgaris* subsp. *maritima* populations and either (1) Euclidian geographical distance matrices, (2) matrices of geographical distance measured along the coastline or (3) geographical distance matrices determined through a neighbourhood graph.

**Table S2.** Linear mixed models with loci as random intercept testing the relationship between genetic diversity parameters (based on nuclear and cytoplasmic polymorphism) and explanatory variables (latitude and coastline distance). Models were performed on two datasets to take into account the genetic discontinuities indicated as G1 for northern populations (labelled 1 to 40 in Fig. 1), and G2 for Moroccan populations at lower latitudes (labelled 43 to *t*).
