# Supplementary material for: Spatial genetic structure in Beta vulgaris subsp. maritima and Beta macrocarpa reveals the effect of contrasting mating system, influence of marine currents, and footprints of postglacial recolonization routes
Source: Ecol Evol. 2014 Apr 18;4(10):1828–52. doi: 10.1002/ece3.1061 (PMC4063479; doi:10.1002/ece3.1061)
Supplement: Supplementary file 1 [file ece30004-1828-SD1.doc]

SUPPLEMENTARY DATA

**Figure S1.** Assignment results from Bayesian clustering following Pritchard *et al*. (2000) performed on **(A)** *B. vulgaris* subsp. *maritima* and *B. macrocarpa* individuals **(B)** *B. vulgaris* subsp. *maritima* individuals only; and, **(C)** following Durand *et al*. (2009) on both species. STRUCTURE analyses **(A and B)**: mean (±s.d.) probabilities of the data Ln Pr(X|*K*) over 15 replicated runs plotted as a function of the putative number of clusters *K* (blue dots) and the standardized second-order rate of change of Ln Pr(X|*K*), *ΔK*, as a function of *K* (red dots). TESS analyses **(c)**: average Deviation Index Criterion (DIC) over 20 replicated runs plotted against *K*.

**Figure S2.** Assignment probabilities of membership of **(A)** *B. vulgaris* subsp. *maritima* individuals into the five inferred clusters for the second modal value, and **(B)** *B. vulgaris* subsp. *maritima and B. macrocarpa* individuals into the six inferred clusters for the second modal value. Each individual is represented by a thin horizontal line (y axis) partitioned into coloured segments that represented the individual’s estimated membership coefficients (x axis) **(c)** Map of mean population membership probabilities for the six clusters.

**Figure S3.** Population genetic structure of *B. vulgaris* subsp. *maritima* inferred from TESS analyses assuming *K*=5. The individual estimates membership coefficients for each cluster are shown for 20 independent runs.

**Figure S4.** Geographical distribution of minisatellites haplotypes within populations.

**Figure S5.** Linear regressions of genetic diversity indices based on nuclear (*A_r_, H*_E_*, A_r_P*) and based on cytoplasmic (*A_r_*) polymorphism with respect to latitude and coastline distance for each locus. Regressions were performed on **(A)** northern populations (labelled *1* to *40* in Figure 1), and **(B)** Moroccan populations at lower latitudes (labelled *43* to *t*).

**Figure S6.** Matrices of population-pairwise differentiation values (*F*_ST_) of *B. vulgaris* subsp. *maritima* and *B. macrocarpa* individuals represented for **(A)** nuclear and **(B)** cytoplasmic data.

**Figure S7.** Individual representation of the three first global axes of the spatial principal component analysis (sPCA) performed on *B. vulgaris* subsp. *maritima* populations. According to the first three global eigenvalues, First **(A)**, second **(B)**, and third **(C)** sPCA scores, are represented on each plot as squares whose size is proportional to the value of the score, so that the maximum differentiation is between large white squares and large black squares.

**Figure S8.** Individual representation of the three first global axes of the spatial principal component analysis (sPCA) performed on *B. vulgaris* subsp. *maritima* and *Beta macrocarpa* populations.

**Figure S9.** Modern (12°C, 14°C and 16°C mean July, northern Europe) and reconstructed (June/July/August isotherms, southern Europe) Last Glacial Maximum (LGM) isotherms from Kadereit *et al*. 2005. Bold lines indicate the ice shield during the LGM and dotted lines indicate the coastline during the LGM.

**Figure S1.**

**
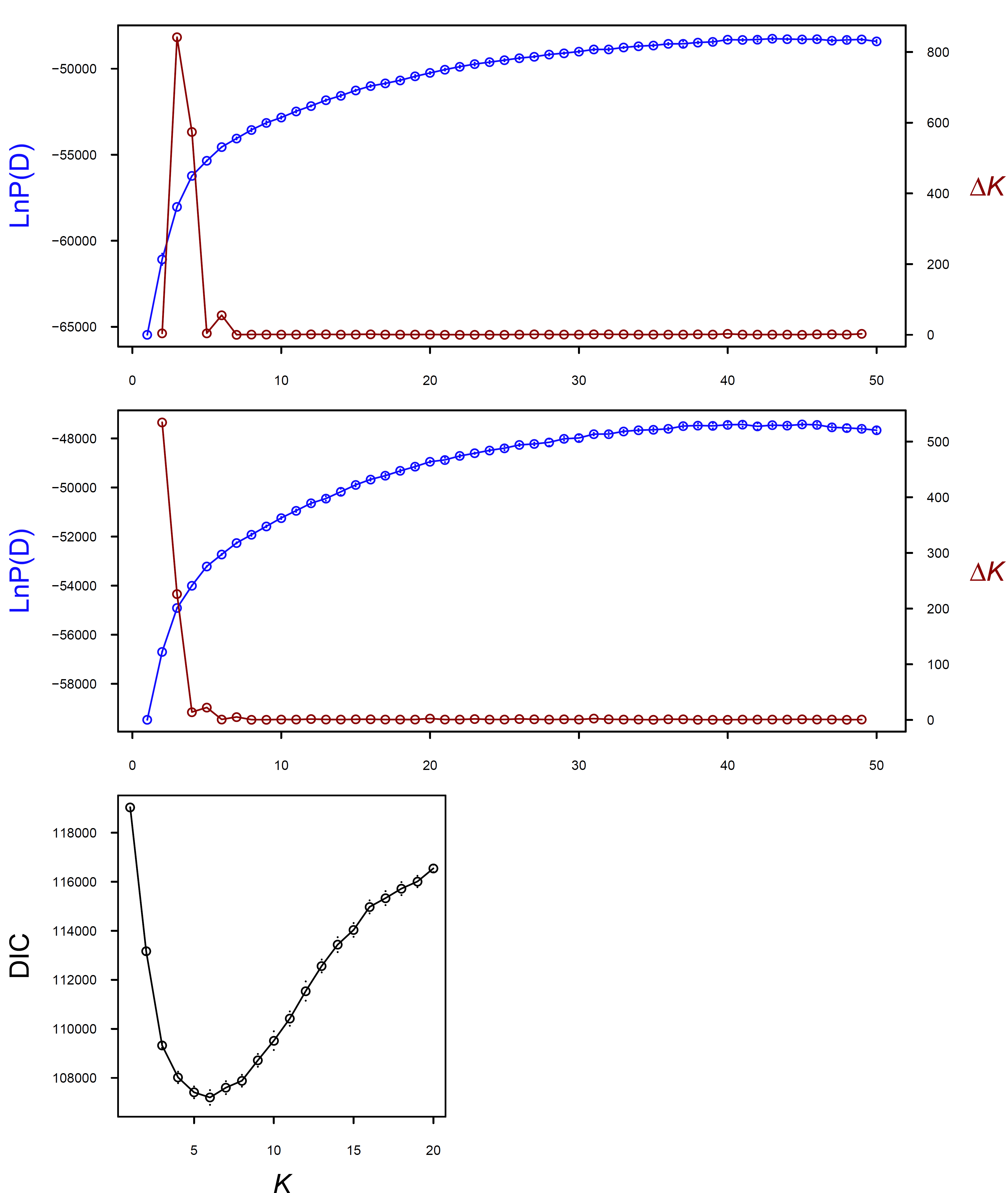
**

**a**

**b**

**c**

**Figure S2.**

**c**

**a b c**

**

**

**Figure S3.**

**

**


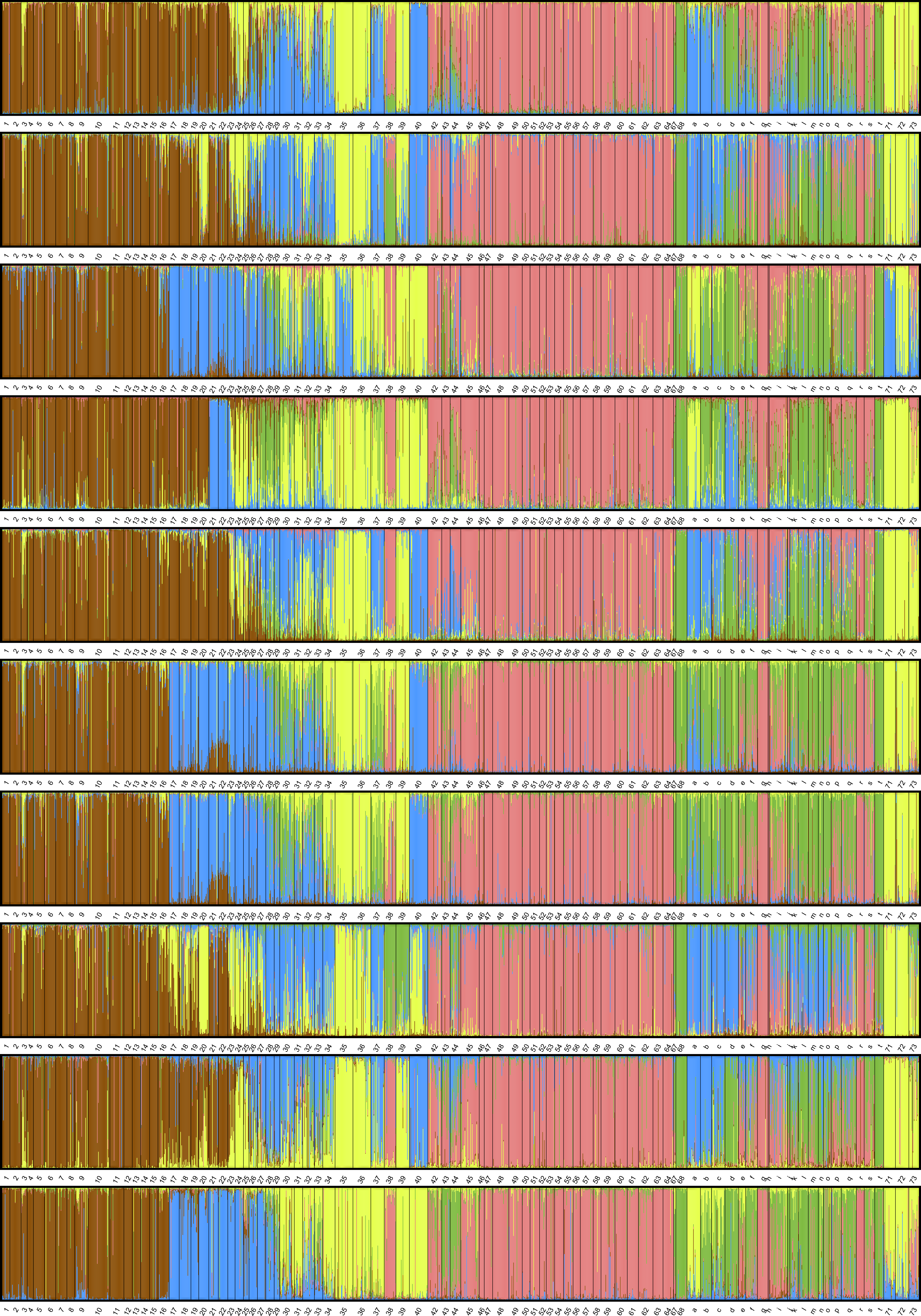
**Figure S3.** (continued)


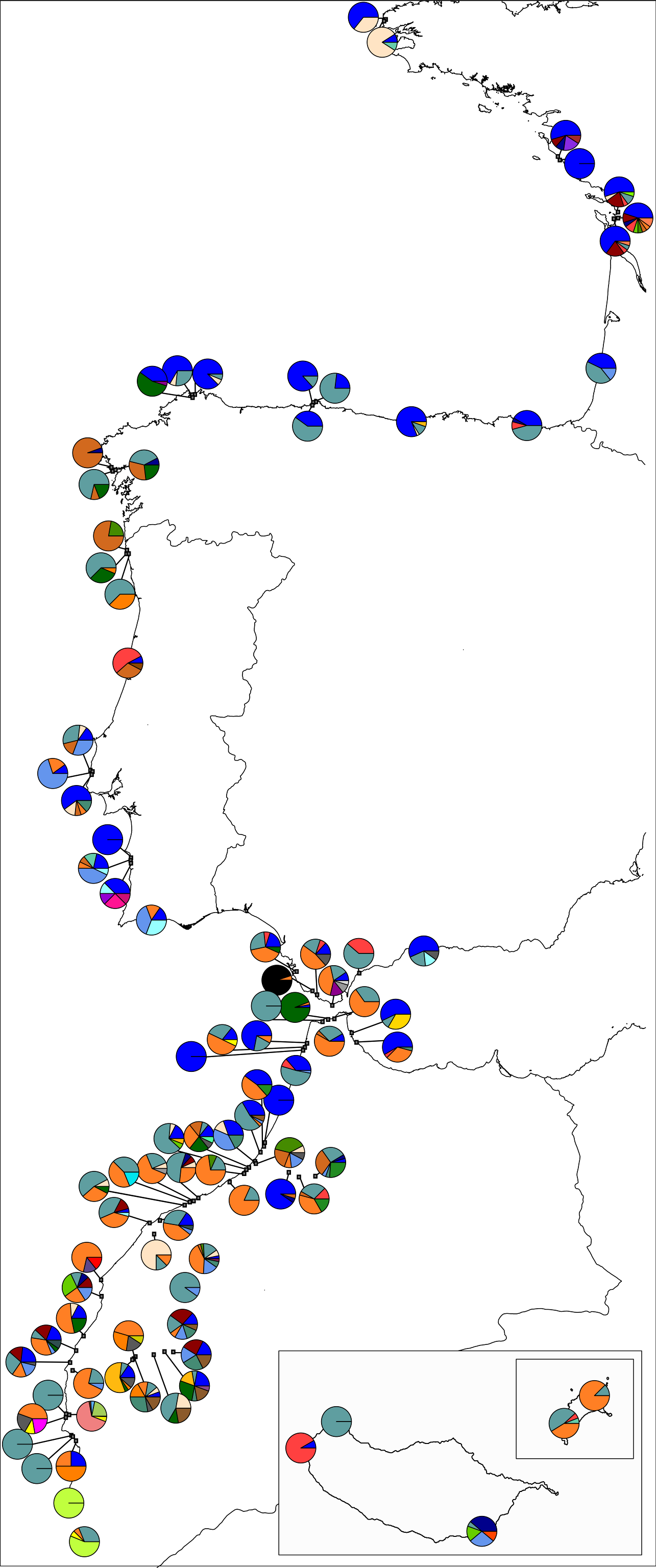
**Figure S4.**

**

Figure S5.**

**a**

**Figure S5.** (continued)

**b**

**

**

**Figure S6.**


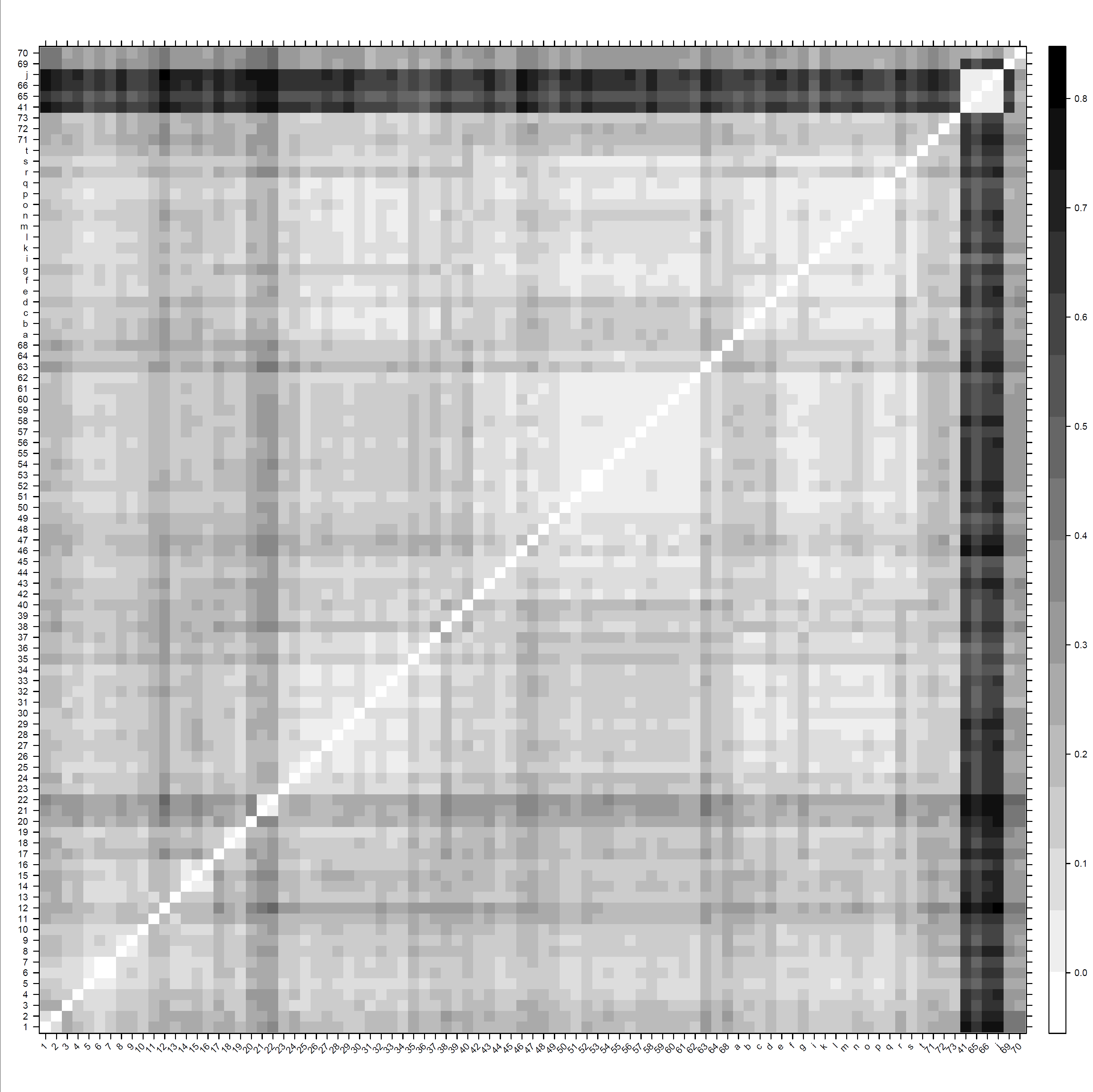

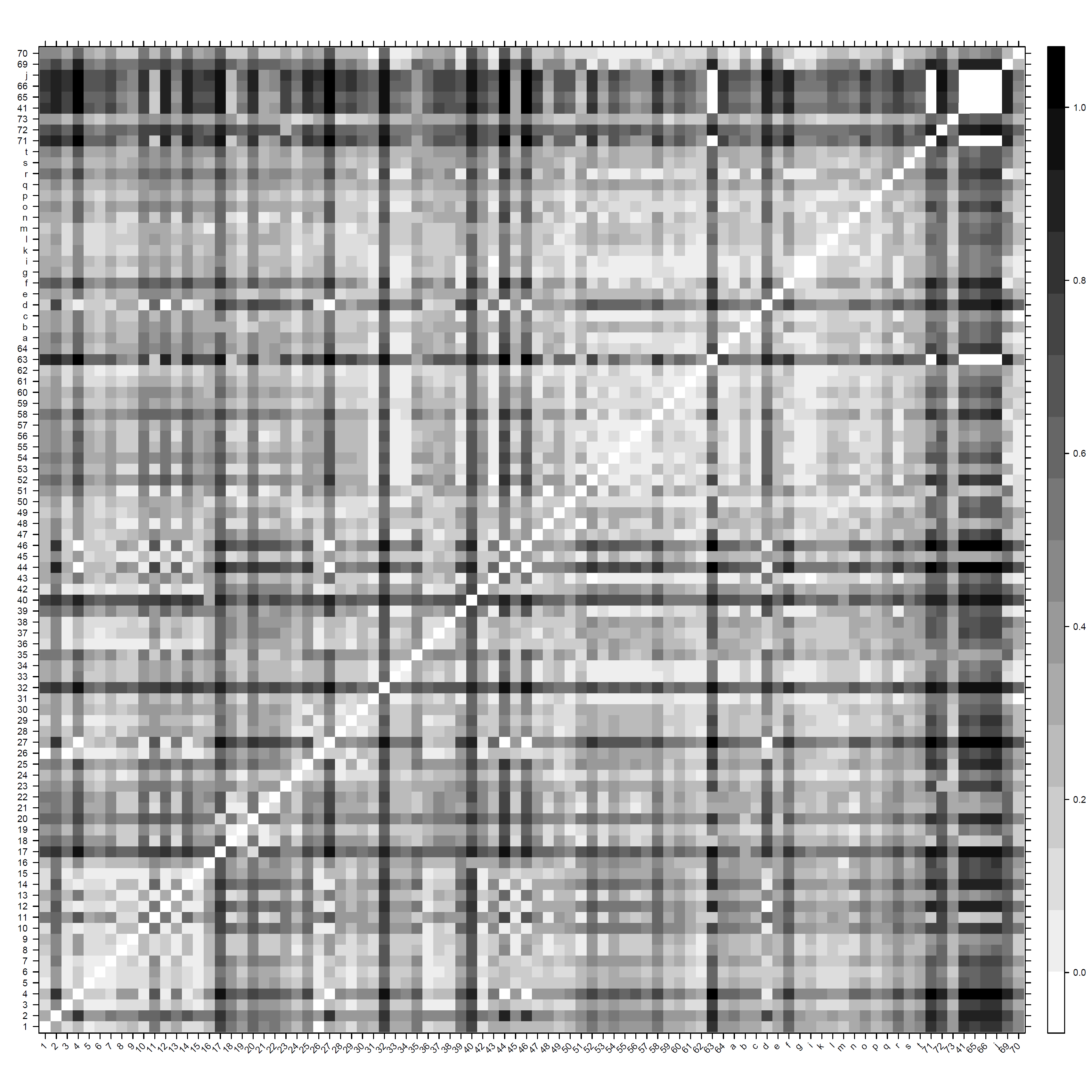


**a**

**b**

**Figure S7.**


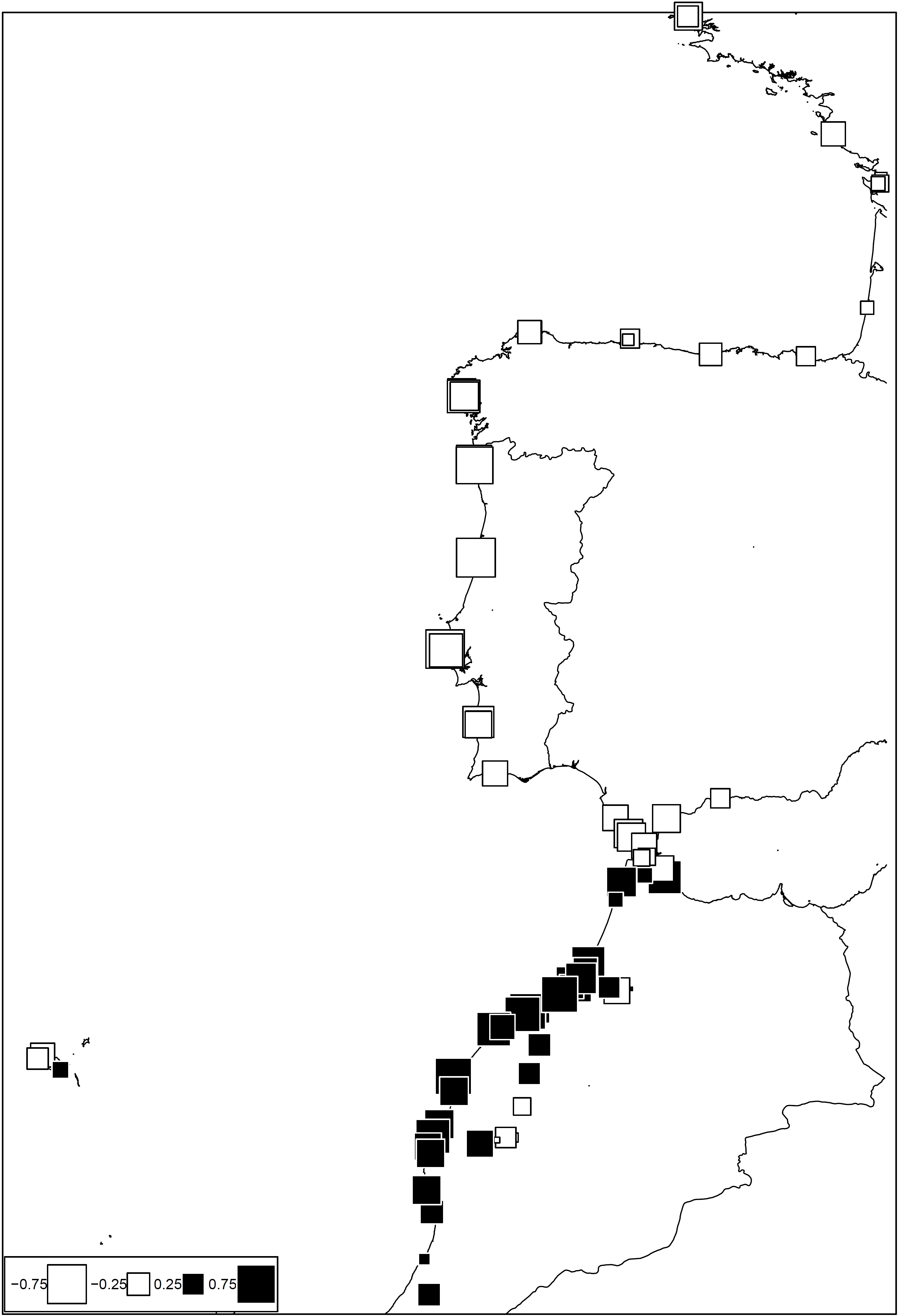

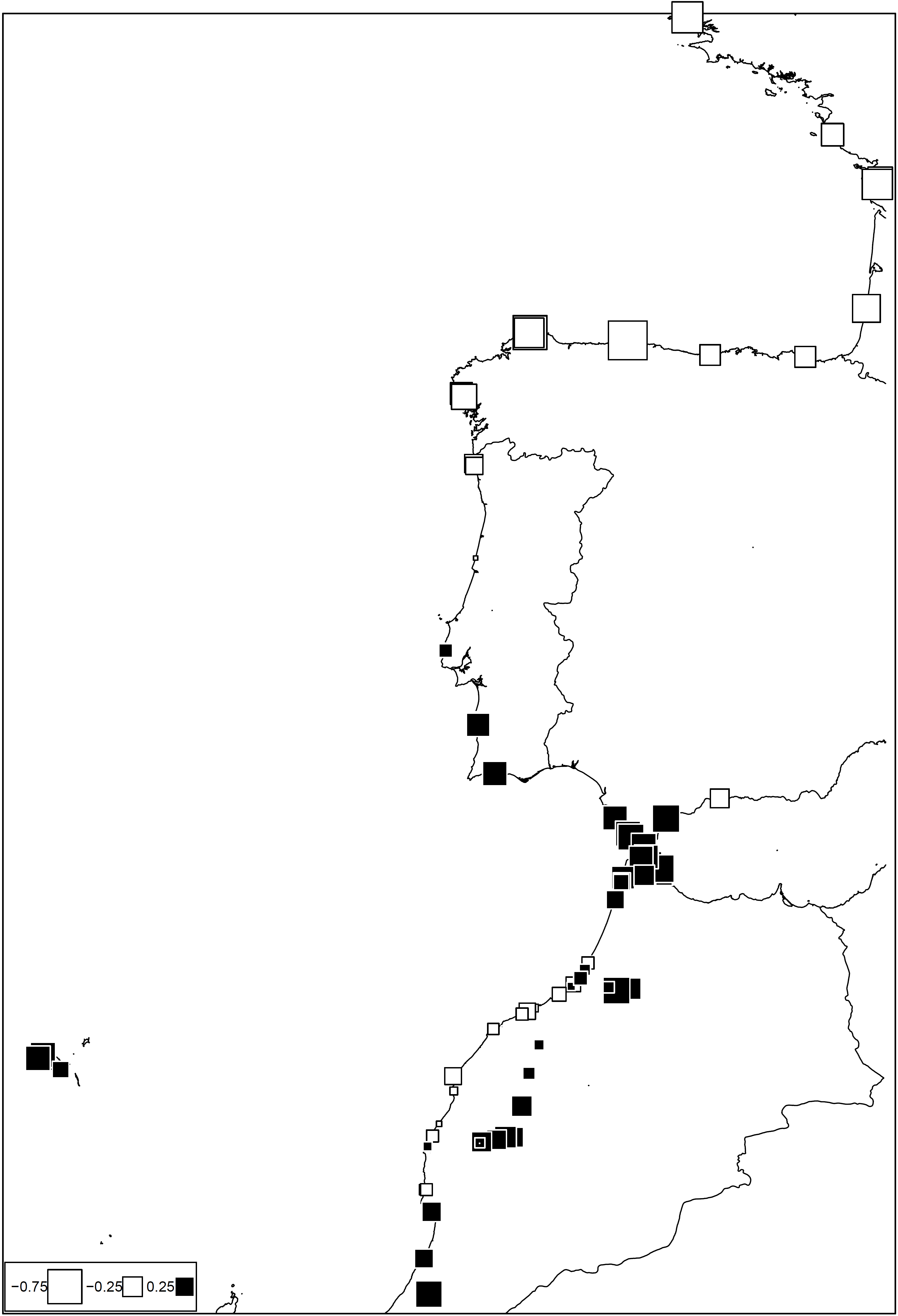

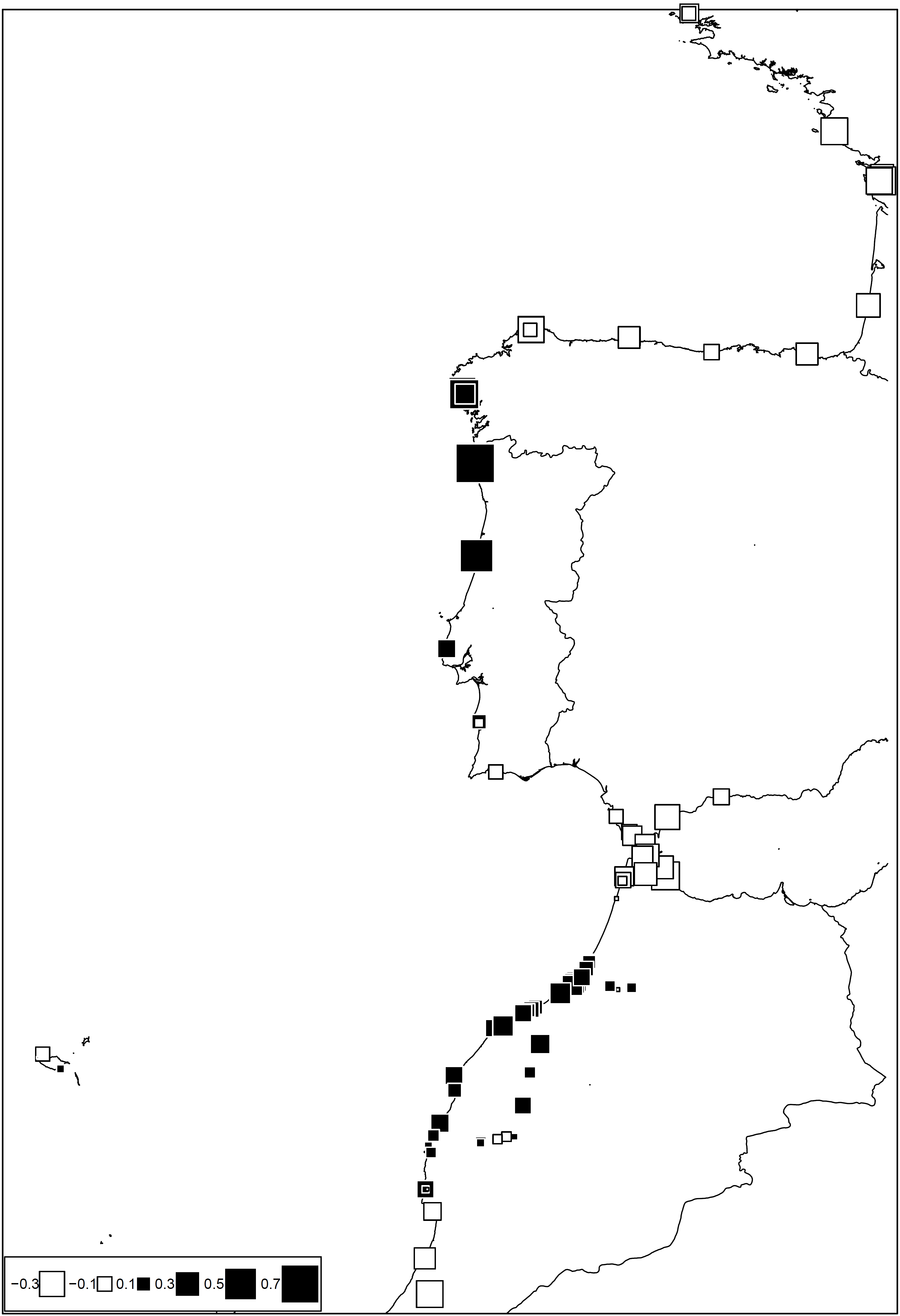


**Figure S8.**

**
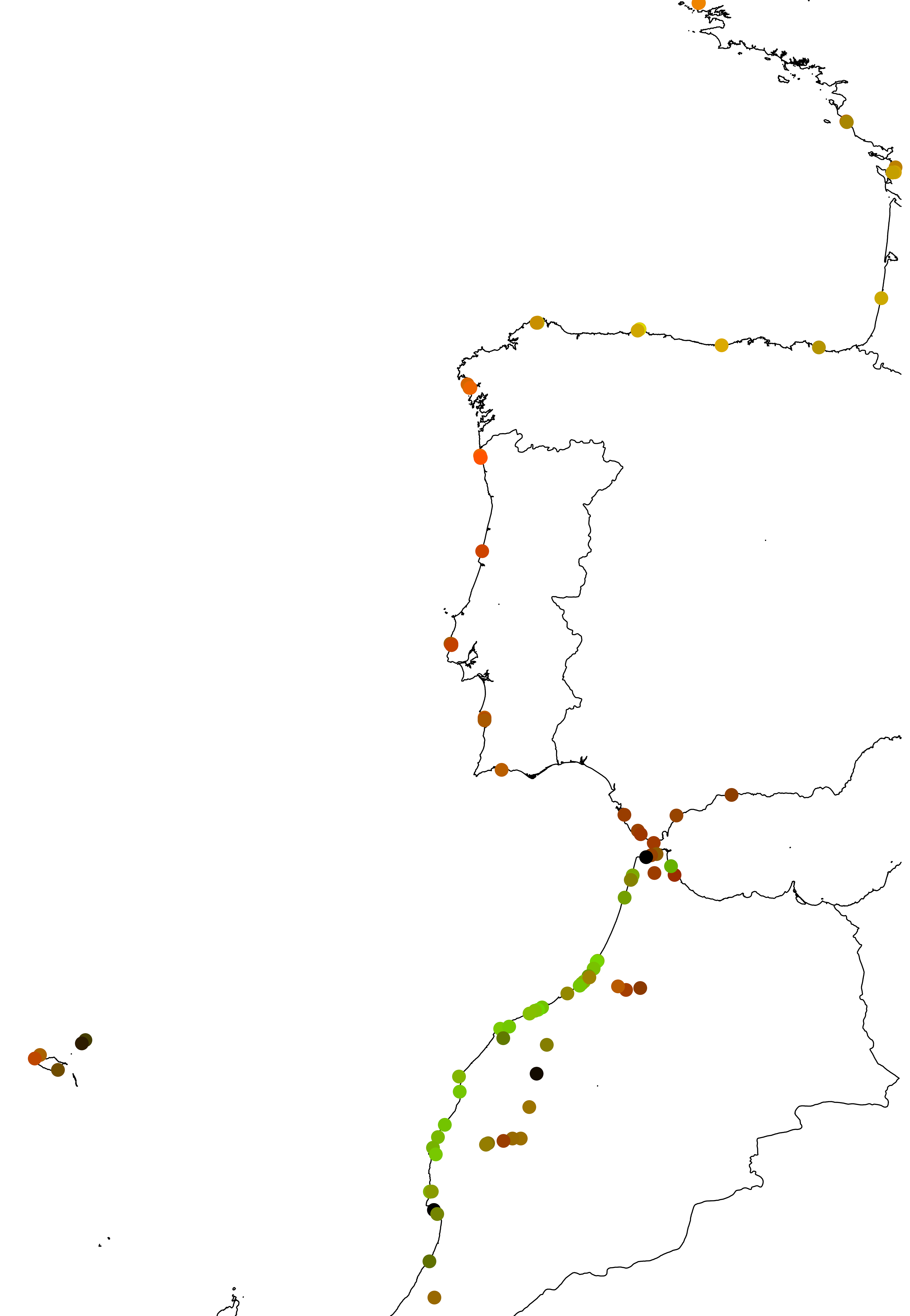
**

**Figure S9.**

**
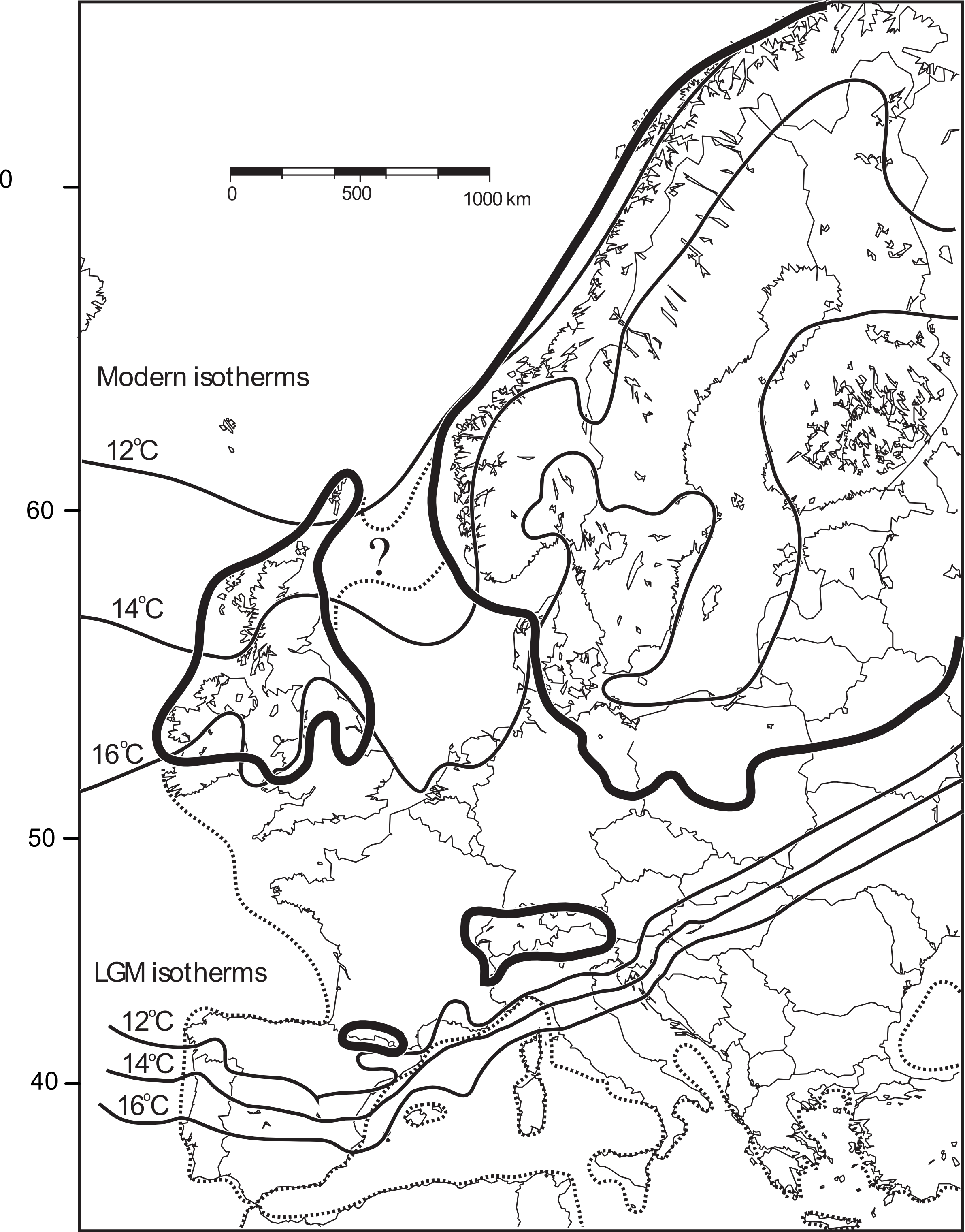
**

**Table S1.** Mantel tests carried out on genetic distance matrices based on nuclear and cytoplasmic data for *B. vulgaris* subsp. *maritima* populations and either (i) Euclidian geographical distance matrices, (ii) matrices of geographical distance measured along the coastline or (iii) geographical distance matrices determined through a neighbourhood graph.

|  | **Nuclear data** | | **Cytoplasmic data** | |
| --- | --- | --- | --- | --- |
|  | *r*^2^ | *P*-value | *r*^2^ | *P*-value |
| **Geographical distance** |  |  |  |  |
| *Euclidean* |  |  |  |  |
| All populations | 0.33232 | 0.001 | 0.08051 | 0.163 |
| All coastal populations (*1* to *67*)* | 0.48968 | 0.001 | 0.08787 | 0.130 |
| Inland ruderal populations (*a* to *t*) | 0.38333 | 0.007 | 0.31159 | 0.008 |
| Coastal populations (*1* to *40*) | 0.50112 | 0.001 | 0.0864 | 0.175 |
| Coastal populations (*42* to *67*) | 0.47686 | 0.001 | 0.25728 | 0.011 |
| *Along the coastline* |  |  |  |  |
| All coastal populations | 0.44369 | 0.001 | 0.04297 | 0.528 |
| **Connection criterion (all populations)** |  |  |  |  |
| Delaunay | 0.33077 | 0.001 | 0.07083 | 0.234 |
| Gabriel | 0.33653 | 0.001 | 0.08683 | 0.128 |
| Relative neighbourhood | 0.30886 | 0.001 | 0.05756 | 0.370 |
| * Madeira populations not included |  |  |  |  |
|  | |  |  |  |

**Table S2.** Linear mixed models with loci as random intercept testing the relationship between genetic diversity parameters (based on nuclear and cytoplasmic polymorphism) and explanatory variables (latitude and coastline distance). Models were performed on two datasets to take into account the genetic discontinuities indicated as G1 for northern populations (labelled *1* to *40* in Figure 1), and G2 for Moroccan populations at lower latitudes (labelled *43* to *t*).

| **G1** | **Nuclear Data** |  |  |  |  |  |
| --- | --- | --- | --- | --- | --- | --- |
|  |  | ***A_r_* ~ Latitude \| Locus** | | | | |
|  |  | ***Random effects:*** ~1\| Locus | (Intercept) | Residual |  |  |
|  |  | StdDev | 1.199 | 1.456 |  |  |
|  |  | ***Fixed effects:*** *A_r_*~ Latitude | **Value** | **Std.Error** | **DF** | **p-value** |
|  |  | (Intercept) | 11.535 | 0.970 | 303 | 0.000 |
|  |  | Latitude | -0.162 | 0.021 | 303 | 0.000 |
|  |  |  |  |  |  |  |
|  |  |  |  |  |  |  |
|  |  | ***A_r_*~ Coastline distance \| Locus** | | | | |
|  |  | ***Random effects:*** ~1\| Locus | (Intercept) | Residual |  |  |
|  |  | StdDev | 1.198 | 1.491 |  |  |
|  |  | ***Fixed effects:*** *A_r_* ~ Coastline distance | **Value** | **Std.Error** | **DF** | **p-value** |
|  |  | (Intercept) | 5.949 | 0.464 | 303 | 0.000 |
|  |  | Coastline distance | - 4.44 10^-4^ | 6.82 10^-5^ | 303 | 0.000 |
|  |  |  |  |  |  |  |
|  |  |  |  |  |  |  |
|  |  | ***H_e_*~ Latitude \| Locus** | | | | |
|  |  | ***Random effects:*** ~1\| Locus | (Intercept) | Residual |  |  |
|  |  | StdDev | 0.091 | 0.162 |  |  |
|  |  | ***Fixed effects:*** *H_e_* ~ Latitude | **Value** | **Std.Error** | **DF** | **p-value** |
|  |  | (Intercept) | 1.264 | 0.102 | 303 | 0.000 |
|  |  | Latitude | -0.015 | 0.002 | 303 | 0.000 |
|  |  |  |  |  |  |  |
|  |  |  |  |  |  |  |
|  |  | ***H_e_*~ Coastline distance \| Locus** | | | | |
|  |  | ***Random effects:*** ~1\| Locus | (Intercept) | Residual |  |  |
|  |  | StdDev | 0.091 | 0.165 |  |  |
|  |  | ***Fixed effects:*** *H_e_*~ Coastline distance | **Value** | **Std.Error** | **DF** | **p-value** |
|  |  | (Intercept) | 0.756 | 0.038 | 303 | 0.000 |
|  |  | Coastline distance | - 4.02 10^-5^ | 7.55 10^-6^ | 303 | 0.000 |
|  |  |  |  |  |  |  |
|  |  |  |  |  |  |  |
|  |  | **A*_r_P* ~ Latitude \| Locus** | | | | |
|  |  | ***Random effects:*** ~1\| Locus | (Intercept) | Residual |  |  |
|  |  | StdDev | 4.283 10^-6^ | 0.120 |  |  |
|  |  | ***Fixed effects:*** *A_r_P* ~ Latitude | **Value** | **Std.Error** | **DF** | **p-value** |
|  |  | (Intercept) | 0.245 | 0.072 | 303 | 0.001 |
|  |  | Latitude | -0.005 | 0.002 | 303 | 0.003 |
|  |  |  |  |  |  |  |
|  |  |  |  |  |  |  |
|  |  | **A*_r_P* ~ Coastline distance \| Locus** | | | | |
|  |  | ***Random effects:*** ~1\| Locus | (Intercept) | Residual |  |  |
|  |  | StdDev | 4.333 10^-6^ | 0.121 |  |  |
|  |  | ***Fixed effects:*** *A_r_P* ~ Coastline distance | **Value** | **Std.Error** | **DF** | **p-value** |
|  |  | (Intercept) | 0.069 | 0.015 | 303 | 0.000 |
|  |  | Coastline distance | - 1.528 10^-5^ | 0.000 | 303 | 0.006 |
|  |  |  |  |  |  |  |
|  | **Cytoplasmic data** |  |  |  |  |  |
|  |  | ***A_r_* ~ Latitude \| Locus** | | | | |
|  |  | ***Random effects:*** ~1\| Locus | (Intercept) | Residual |  |  |
|  |  | StdDev | 0.870 | 0.829 |  |  |
|  |  | ***Fixed effects:*** *A_r_*~ Latitude | **Value** | **Std.Error** | **DF** | **p-value** |
|  |  | (Intercept) | 2.811 | 0.826 | 151 | 0.001 |
|  |  | Latitude | -0.023 | 0.017 | 151 | 0.172 |
|  |  |  |  |  |  |  |
|  |  |  |  |  |  |  |
|  |  | ***A_r_*~ Coastline distance \| Locus** | | | | |
|  |  | ***Random effects:*** ~1\| Locus | (Intercept) | Residual |  |  |
|  |  | StdDev | 0.870 | 0.829 |  |  |
|  |  | ***Fixed effects:*** *A_r_* ~ Coastline distance | **Value** | **Std.Error** | **DF** | **p-value** |
|  |  | (Intercept) | 2.018 | 0.460 | 151 | 0.000 |
|  |  | Coastline distance | 0.000 | 0.000 | 151 | 0.211 |
|  |  |  |  |  |  |  |
| **G2** | **Nuclear Data** |  |  |  |  |  |
|  |  | ***A_r_* ~ Latitude \| Locus** | | | | |
|  |  | ***Random effects:*** ~1\| Locus | (Intercept) | Residual |  |  |
|  |  | StdDev | 2.603 | 1.458 |  |  |
|  |  | ***Fixed effects:*** *A_r_*~ Latitude | **Value** | **Std.Error** | **DF** | **p-value** |
|  |  | (Intercept) | 4.044 | 2.375 | 191 | 0.090 |
|  |  | Latitude | 0.045 | 0.066 | 191 | 0.490 |
|  |  |  |  |  |  |  |
|  |  |  |  |  |  |  |
|  |  | ***A_r_*~ Coastline distance \| Locus** | | | | |
|  |  | ***Random effects:*** ~1\| Locus | (Intercept) | Residual |  |  |
|  |  | StdDev | 2.603 | 1.459 |  |  |
|  |  | ***Fixed effects:*** *A_r_* ~ Coastline distance | **Value** | **Std.Error** | **DF** | **p-value** |
|  |  | (Intercept) | 5.447 | 0.952 | 191 | 0.000 |
|  |  | Coastline distance | 0.000 | 0.000 | 191 | 0.624 |
|  |  |  |  |  |  |  |
|  |  |  |  |  |  |  |
|  |  | ***H_e_*~ Latitude \| Locus** | | | | |
|  |  | ***Random effects:*** ~1\| Locus | (Intercept) | Residual |  |  |
|  |  | StdDev | 0.226 | 0.109 |  |  |
|  |  | ***Fixed effects:*** *H_e_* ~ Latitude | **Value** | **Std.Error** | **DF** | **p-value** |
|  |  | (Intercept) | 0.405 | 0.183 | 191 | 0.028 |
|  |  | Latitude | 0.008 | 0.005 | 191 | 0.114 |
|  |  |  |  |  |  |  |
|  |  |  |  |  |  |  |
|  |  | ***H_e_*~ Coastline distance \| Locus** | | | | |
|  |  | ***Random effects:*** ~1\| Locus | (Intercept) | Residual |  |  |
|  |  | StdDev | 0.226 | 0.110 |  |  |
|  |  | ***Fixed effects:*** *H_e_*~ Coastline distance | **Value** | **Std.Error** | **DF** | **p-value** |
|  |  | (Intercept) | 0.642 | 0.082 | 191 | 0.000 |
|  |  | Coastline distance | 0.000 | 0.000 | 191 | 0.162 |
|  |  |  |  |  |  |  |
|  |  |  |  |  |  |  |
|  |  | **A*_r_P* ~ Latitude \| Locus** | | | | |
|  |  | ***Random effects:*** ~1\| Locus | (Intercept) | Residual |  |  |
|  |  | StdDev | 0.043 | 0.194 |  |  |
|  |  | ***Fixed effects:*** *A_r_P* ~ Latitude | **Value** | **Std.Error** | **DF** | **p-value** |
|  |  | (Intercept) | -0.189 | 0.292 | 191 | 0.518 |
|  |  | Latitude | 0.007 | 0.009 | 191 | 0.396 |
|  |  |  |  |  |  |  |
|  |  |  |  |  |  |  |
|  |  | **A*_r_P* ~ Coastline distance \| Locus** | | | | |
|  |  | ***Random effects:*** ~1\| Locus | (Intercept) | Residual |  |  |
|  |  | StdDev | 0.043 | 0.194 |  |  |
|  |  | ***Fixed effects:*** *A_r_P* ~ Coastline distance | **Value** | **Std.Error** | **DF** | **p-value** |
|  |  | (Intercept) | 0.035 | 0.036 | 191 | 0.338 |
|  |  | Coastline distance | 0.000 | 0.000 | 191 | 0.414 |
|  |  |  |  |  |  |  |
|  | **Cytoplasmic data** |  |  |  |  |  |
|  |  | ***A_r_* ~ Latitude \| Locus** | | | | |
|  |  | ***Random effects:*** ~1\| Locus | (Intercept) | Residual |  |  |
|  |  | StdDev | 0.986 | 0.761 |  |  |
|  |  | ***Fixed effects:*** *A_r_*~ Latitude | **Value** | **Std.Error** | **DF** | **p-value** |
|  |  | (Intercept) | 4.925 | 1.898 | 91 | 0.011 |
|  |  | Latitude | -0.091 | 0.055 | 91 | 0.098 |
|  |  |  |  |  |  |  |
|  |  |  |  |  |  |  |
|  |  | ***A_r_*~ Coastline distance \| Locus** | | | | |
|  |  | ***Random effects:*** ~1\| Locus | (Intercept) | Residual |  |  |
|  |  | StdDev | 0.986 | 0.762 |  |  |
|  |  | ***Fixed effects:*** *A_r_* ~ Coastline distance | **Value** | **Std.Error** | **DF** | **p-value** |
|  |  | (Intercept) | 2.174 | 0.534 | 91 | 0.000 |
|  |  | Coastline distance | -0.001 | 0.000 | 91 | 0.105 |
|  |  |  |  |  |  |  |
